# DUB3/KLF4 combats tumor growth and chemoresistance in hepatocellular carcinoma

**DOI:** 10.1038/s41420-022-00988-5

**Published:** 2022-04-05

**Authors:** Xuebing Jia, Lei Li, Fan Wang, Ying Xue, Tongde Wu, Qianqian Jia, Yunhui Li, Chenming Wu, Yuping Chen, Jinhuan Wu, Yang Su, Xinshu Wang, Tao Zhuang, Xiao Dong, Jing Ling, Jian Yuan, Qi Li

**Affiliations:** 1 Cancer Center, Shanghai General Hospital, Shanghai Jiao Tong University School of Medicine, Shanghai, 200080 China; 2 Key Laboratory of Arrhythmias of the Ministry of Education of China, Research Center for Translational Medicine, East Hospital, Tongji University School of Medicine, Shanghai, 200120 China; 3 Department of Biochemistry and Molecular Biology, Tongji University School of Medicine, Shanghai, 200120 China; 4 Zhongshan Hospital Institute of Clinical Science, Fudan University, Shanghai, 200032 China; 5 Department of Spine Surgery, Shanghai East Hospital, Tongji University School of Medicine, Shanghai, 200120 China; 6 CAS Key Laboratory of Tissue Microenvironment and Tumor, Shanghai Institute of Nutrition and Health, Shanghai Institutes for Biological Sciences, Chinese Academy of Sciences, University of Chinese Academy of Sciences, Shanghai, 200031 China; 7 Basic Medicine College, Jinzhou Medical University, Liaoning, 121000 China

**Keywords:** Cancer, Liver cancer

## Abstract

This study aimed to investigate the role of deubiquitinating enzyme 3 (DUB3) in the regulation of Krüppel-like factor 4 (KLF4) expression in hepatocellular carcinoma (HCC). Gain- and loss-of-function assay, luciferase reporter assay, co-immunoprecipitation, and intracellular and extracellular deubiquitination assays were conducted in vitro. A tumor xenograft mouse model was established. The expression of DUB3 and KLF4 was examined in HCC patient specimens. The results showed that DUB3 upregulated KLF4 expression by deubiquitinating and stabilizing KLF4 protein in HCC cells through binding with KLF4. DUB3 inhibited HCC cell proliferation in vitro and tumor growth in vivo while enhancing the chemosensitivity of HCC cells in a KLF4-dependent manner. Furthermore, KLF4 promoted DUB3 transcription by binding to the DUB3 promoter. In HCC patients, DUB3 expression positively correlated with KLF4 expression in HCC tissues. Low DUB3 expression predicted worse overall survival and recurrence in HCC patients. In conclusion, this study revealed a positive DUB3/KLF4 feedback loop that inhibits tumor growth and chemoresistance in HCC. These results suggest that DUB3/KLF4 activation might be a potential therapeutic approach for HCC treatment.

## Introduction

Hepatocellular carcinoma (HCC) is the fifth and seventh leading cause of cancer-related deaths in men and women, respectively, worldwide [1, 2]. Despite the advances in systemic therapy, the mortality of HCC remains high [3–5]. Combination therapies have shown remarkable therapeutic effects compared with single agents in HCC treatment; however, these therapies are associated with high toxicity, and chemoresistance can develop [6]. Therefore, it is urgently needed to better understand the underlying mechanisms of HCC pathogenesis and chemoresistance and to identify new therapeutic targets for HCC treatment.

Krüppel-like factor 4 (KLF4) is a highly conserved eukaryotic transcription factor involved in cell proliferation, differentiation, pluripotency, and embryonic development [7, 8]. KLF4 is differentially expressed in different types of cancer [9]. Studies have shown that KLF4 acts as a tumor suppressor in colorectal cancer, gastric cancer, and bladder cancer [10–12] while playing an oncogenic role in breast cancer and skin cancer [13, 14]. KLF4 is significantly downregulated in HCC and inhibits HCC cell proliferation, migration, and invasion by upregulating the expression of genes involved in mesenchymal-epithelial transformation (MET) [15]. Ubiquitin-mediated degradation of KLF4 plays a pivotal role in promoting HCC progression. For example, F-box only protein 22 promotes HCC proliferation through ubiquitination and degradation of KLF4 [16]. TRAF7 enhances ubiquitin-mediated degradation of KLF4 to promote HCC progression [17]. FBXO32 suppresses breast cancer tumorigenesis by targeting KLF4 to proteasomal degradation [18]. However, the deubiquitination process of KLF4 remains unclear. After screening a panel of deubiquitination enzymes (DUBs), we found that DUB3 could deubiquitinate and stabilize KLF4 in HCC cells. This preliminary finding prompts us to investigate the DUB3/KLF4 interaction in HCC.

DUB3 plays a significant role in multiple biological processes, such as DNA damage response [19], MET [20, 21], cell proliferation [22], invasion [20], and cell-cycle progression [23–26]. The CDK4/6–DUB3 axis promotes breast cancer metastasis, serving as a promising therapeutic target for the treatment of breast cancer [27]. These findings suggest that DUB3 is a tumor promoter and a potential therapeutic target for cancer treatment.

In the present study, we explored the regulatory role of DUB3 in the expression and stability of KLF4 protein in HCC cells. Knockdown of DUB3 and KLF4 was performed to examine the role and underlying mechanism of DUB3 in cell proliferation, chemoresistance, and xenograft tumor growth in HCC. We also investigated the physical and functional interaction between DUB3 and KLF4 in HCC cells and evaluated the clinical significance of the DUB3/KLF4 axis in HCC patients.

## Results

### DUB3 upregulates KLF4 expression and stabilizes KLF4 protein in HCC cells

**Fig. 1:**
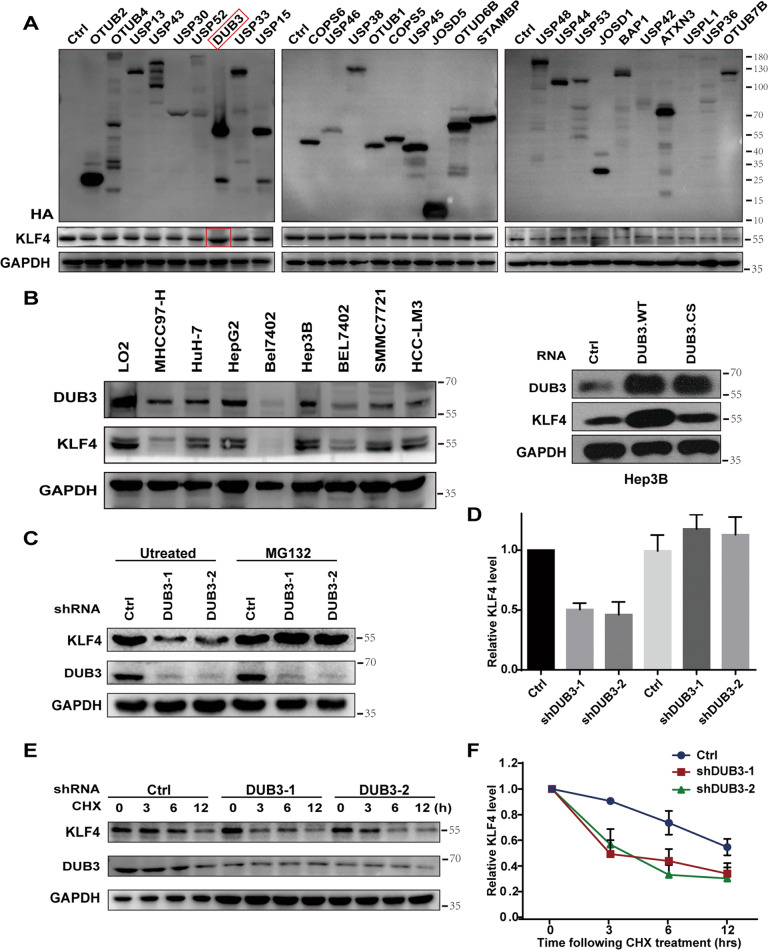
Deubiquitinating enzyme 4 (DUB3) upregulated Krüppel-like factor 4 (KLF4) expression and stabilized KLF4 protein in hepatocellular carcinoma (HCC) cells. **A** HEK293T cells were transfected with deubiquitinase-overexpressing vectors (HA-tag) as indicated. Western blot analysis was performed to detect the protein expression of KLF4. **B** Left panel: The cell lysates from HCC cell lines were blotted for DUB3 and KLF4 expression. The cell lysates from normal liver (LO2) cells were used as a control. Right panel: Hep3B cells were transfected with wild-type DUB3- and C89S mutant-overexpressing vectors. The cell lysates were collected and blotted for DUB3 and KLF4 expression. GAPDH was used as an internal reference. **C**, **D** Hep3B cells stably expressing control (ctrl) or DUB3 shRNA (shDUB3) were incubated with vehicle or MG132 (25 μM) for 5 h. Western blot analysis was conducted to determine the protein expression of KLF4 and DUB3. **E**, **F** Hep3B cells stably expressing ctrl shRNA or shDUB3 were treated with 0.1 mg/mL cycloheximide for 0, 3, 6, or 12 h. Western blot analysis was conducted to determine the protein expression of KLF4 and DUB3. Data are expressed as the mean ± standard error of the mean (SEM). *P < 0.05, **P < 0.01, ***P < 0.001, vs. Ctrl; *n* = 3.

The overexpression of DUB3 significantly upregulated protein expression of KLF4 compared with other DUBs (Fig.1A and Supplementary Fig. 1A). Western blot analysis showed that the protein levels of both DUB3 and KLF4 were considerably decreased in multiple HCC cell lines compared with those in LO2 cells (Fig. 1B, left panel). Overexpression of wild-type DUB3 but not DUB3 mutant enhanced KLF4 protein expression in Hep3B cells (Fig. 1B, right panel). In contrast, knockdown of DUB3 significantly attenuated KLF4 protein expression in HepG2 cells, which was entirely reversed by proteasome inhibitor MG132 (Fig. 1C, D). Knockdown of DUB3 did not change KLF4 mRNA expression in Hep3B cells (Supplementary Fig. 1B). In addition, compared with negative control, knockdown of DUB3 resulted in remarkable reductions in KLF4 protein levels in response to translation inhibitor cycloheximide in a time-dependent manner (Fig. 1E, F).

### DUB3 physically interacts with KLF4

**Fig. 2:**
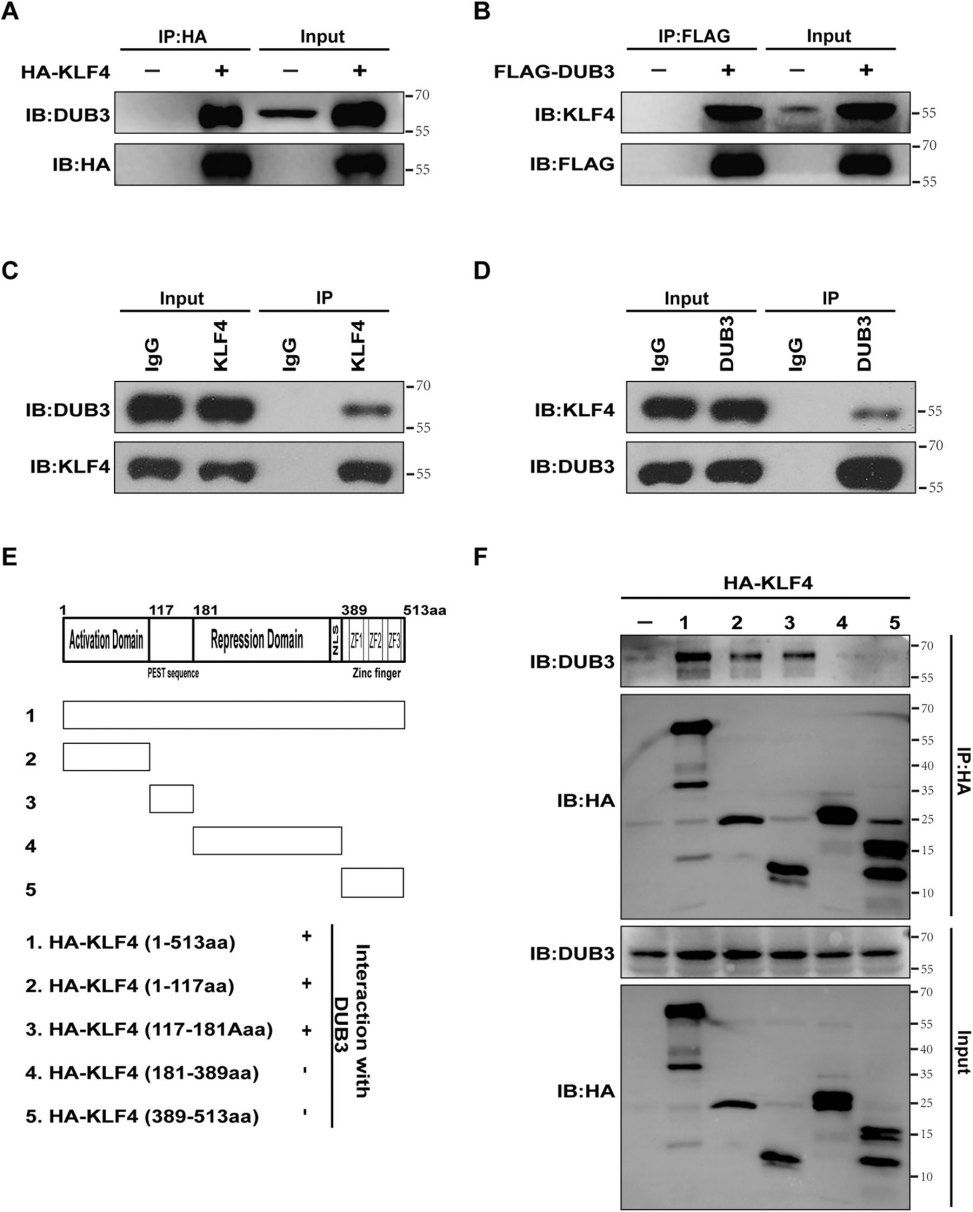
Physical interaction between DUB3 and KLF4 proteins. **A** HEK293T cells were transfected with hemagglutinin (HA)-KLF4. Cell lysates were incubated with anti-HA-agarose, followed by Western blot analysis to detect DUB3 expression. **B** HEK293T cells were transfected with FLAG-DUB3. Cell lysates were incubated with anti-FLAG-agarose, followed by Western blot analysis to detect KLF4 expression. **C**, **D** Cell lysates of Hep3B cells were immunoprecipitated with control IgG, anti-KLF4, or anti-DUB3, as indicated, followed by Western blot analysis to detect DUB3 and KLF4 expression. **E** A schematic diagram of KLF4 domains and deletion mutations. **F** The plasmids expressing KLF mutants (**E**) were transfected into HEK293T cells. Cell lysates were incubated with anti-HA-agarose, followed by Western blot analysis to detect DUB3 expression.

The Co-IP assay revealed physical interactions between DUB3 and HA-tagged KLF4, KLF4, and FLAG-tagged DUB3, as well as endogenous DUB3 and KLF4 in HEK293T cells and Hep3B cells (Fig. 2A–D). The results of the Co-IP assay showed that 117–181 and 181–389 residues of KLF4 were both essential for the binding of DUB3 (Fig. 2E, F).

### DUB3 deubiquitinates KLF4 protein

**Fig. 3:**
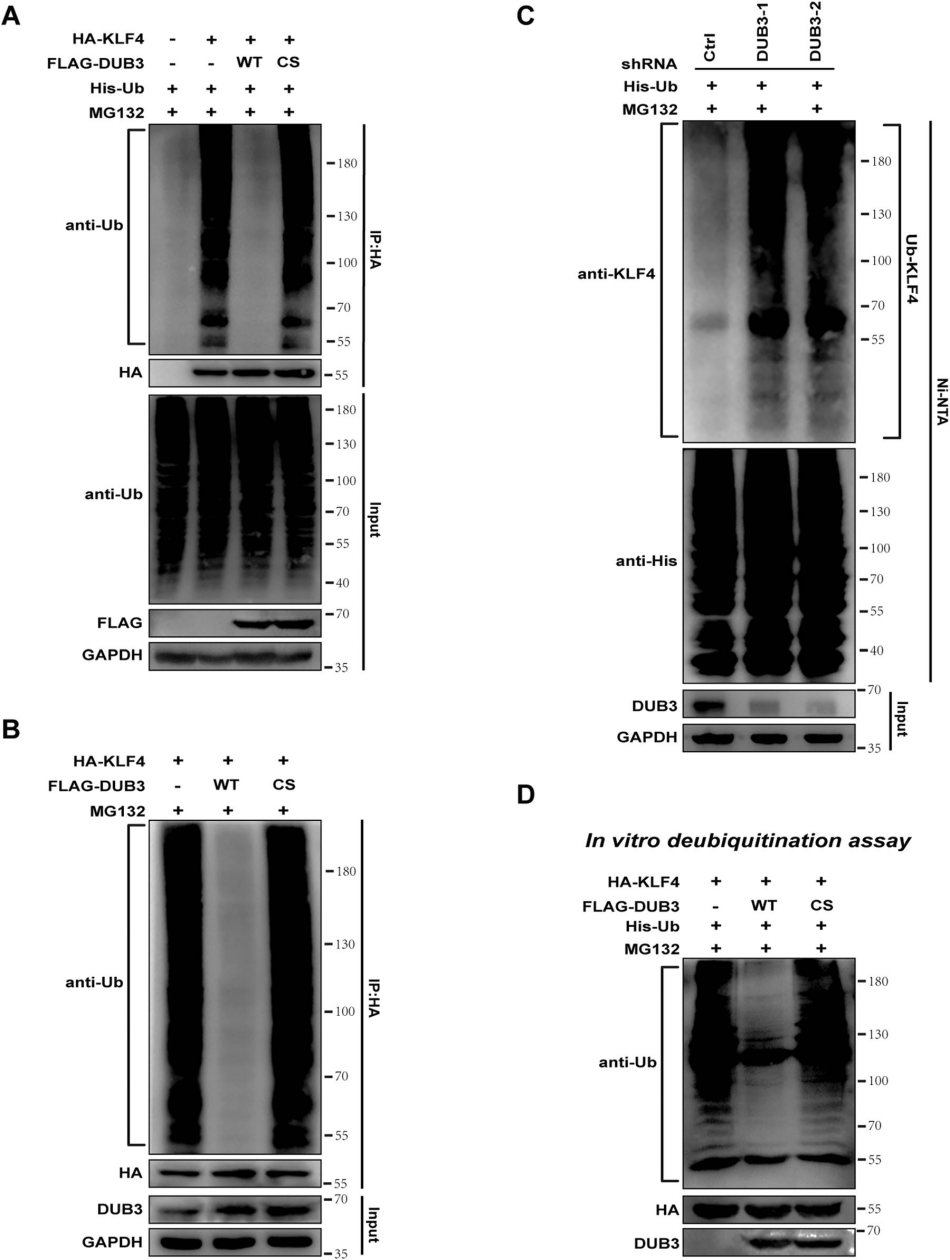
DUB3 deubiquitinated KLF4 intracellularly and extracellularly. **A** HEK293T cells were transfected with vectors expressing HA-KLF4, wild-type FLAG-DUB3, FLAG-DUB3 mutant, or His-ubiquitin (Ub) and treated with 25 μM MG132 for 5 h before collection. Cell lysates were immunoprecipitated with HA-tagged beads and immunoblotted to detect ubiquitin expression. **B** Hep3B cells were cotransfected with vectors expressing HA-KLF4 and wild-type FLAG-DUB3 or FLAG-DUB3 mutant and treated with 25 μM MG132 for 5 h before collection. Cell lysates were immunoprecipitated with HA-tagged beads and immunoblotted to detect ubiquitin expression. **C** Hep3B cells stably expressing control or DUB3 shRNAs were transfected with vectors expressing His-Ub and treated with 25 μM MG132 for 5 h before collection. Cell lysates were immunoprecipitated with Ni-NTA beads and immunoblotted to detect KLF4 expression. **D** HEK293T cells were cotransfected with HA-KLF4 and His-Ub. The ubiquitinated-KLF4 proteins were purified from the cell lysates under the denatured condition and incubated with wild-type DUB3 or DUB3 mutant. The complexes were blotted to detect ubiquitin, HA, and DUB3 expression.

The results of the intracellular deubiquitination assay showed that overexpression of wild-type DUB3 but not DUB3 C89S mutant resulted in a dramatic decrease in ubiquitinated-HA-KLF4 in HA-KLF4-overexpressing HEK293T (Fig. 3A) and Hep3B (Fig. 3B) cells in the presence of MG132. Conversely, knockdown of DUB3 substantially increased ubiquitinated-KLF4 in Hep3B cells in the presence of MG132, compared with control (Fig. 3C).

We cotransfected HA-KLF4 and His-Ub into HEK293T cells and purified the ubiquitinated-KLF4 under the denatured condition. After incubating wild-type DUB3 or DUB3 C89S mutant with the ubiquitinated-KLF4, we found that wild-type DUB3 but not the mutant deubiquitinated KLF4 outside the cells (Fig. 3D).

### DUB3 promotes cell proliferation and tumor growth in HCC via suppressing KLF4 expression

**Fig. 4:**
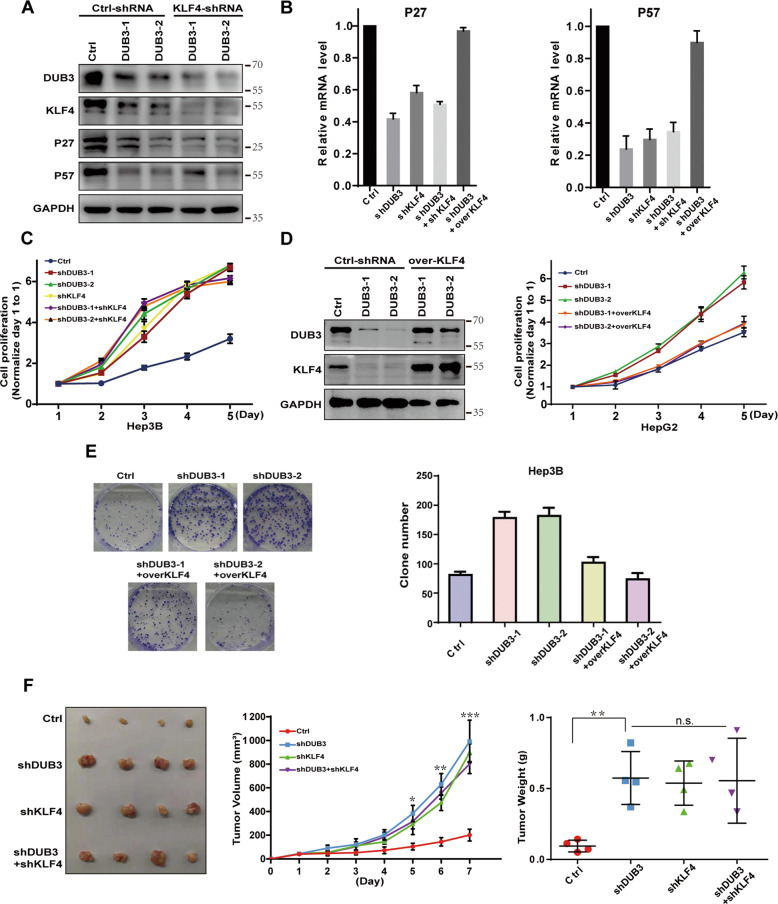
Knockdown of DUB3 promoted HCC cell proliferation and tumor growth via suppressing KLF4 expression. **A** Hep3B cells stably expressing control or the indicated shRNAs were harvested and lysed. Western blotting was carried out to determine protein expression of DUB3, KLF4, P27, and P57. **B** Quantitative real-time PCR (qRT-qPCR) was performed to measure mRNA levels of P27 and P57 in Hep3B cells stably expressing the shRNAs or KLF4 as indicated. **C** Hep3B cells were transfected with shDUB3 and shKLF4 alone or in combination. CCK8 assay was performed to examine cell proliferation at 1, 2, 3, 4, and 5 days after transfection. **D** HepG2 cells stably expressing shDUB3 were transfected with control or vectors expressing KLF4. Western blot analysis was carried out to detect DUB3 and KLF4 expression (left panel). CCK8 assay was performed to examine cell proliferation at 1, 2, 3, 4, and 5 days after transfection (right panel). Data are expressed as the mean ± SEM. *P < 0.05, **P < 0.01, ***P < 0.001, vs. Ctrl; *n* = 3. **E** Hep3B cells were transfected with shDUB3 alone or in combination with KLF4, colony formation assay was used for cell proliferation analysis. **F** Hep3B cells (2 × 10^6^) cells transfected with shDUB3 and shKLF4 alone or in combination were implanted subcutaneously in BALB/c nude mice. The volumes of tumors were measured daily. Tumors were collected 4 weeks after inoculation. Tumor images are shown. Data are expressed as the mean ± standard deviation. *P < 0.05, **P < 0.01, ***P < 0.001, vs. Ctrl; ns, non-significant; *n* = 4.

Western blot analysis revealed that knockdown of DUB3 in Hep3B cells significantly reduced protein levels of KLF4 and KLF4 target genes P27 and P57 that are negatively correlated with HCC cell proliferation [28] (Fig. 4A). In addition, knockdown of DUB3 and KLF4 alone or in combination significantly suppressed the transcription of P27 and P57 in Hep3B cells, compared with negative control. Importantly, overexpression of KLF4 restored P27 and P57 transcription suppressed by DUB3 silencing (Fig. 4B).

Compared with control, knockdown of DUB3 and KLF4 alone or in combination notably promoted Hep3B cell proliferation. However, combined knockdown of DUB3 and KLF4 did not further promote cell proliferation compared with knockdown of DUB3 or KLF4 alone (Fig. 4C). Of note, overexpression of KLF4 abolished the promoting effect of DUB3 silencing on HepG2 (Fig. 4D) and Hep3B (Fig. 4E) cell proliferation.

We examined the role of DUB3 in HCC tumor growth in vivo. As shown in Fig. 4F and Supplementary Fig. 1D, compared with the control, knockdown of DUB3 and KLF4 alone or in combination markedly promoted tumor growth and resulted in remarkably increased tumor volumes and tumor weights in mice at four weeks after tumor cell implantation. However, combined knockdown of DUB3 and KLF4 did not further promote tumor growth compared with knockdown of DUB3 or KLF4 alone.

### DUB3 enhances the chemosensitivity of HCC cells via KLF4

**Fig. 5:**
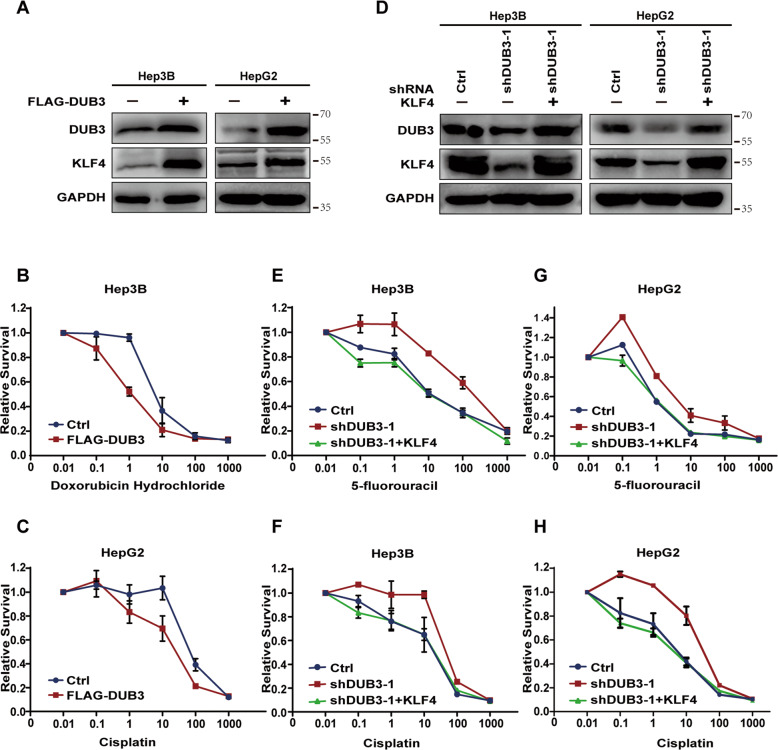
DUB3 enhanced chemosensitivity of HCC cells via KLF4. Hep3B and HepG2 cells were transiently transfected with the plasmids as indicated. **A**, **D** Western blot analysis was performed to determine protein expression of DUB3 and KLF4. **B**, **C**, **E**–**H** The transfected Hep3B and HepG2 cells were treated with different doses of hydrochloride, 5-fluorouracil, or cisplatin for 48 or 72 h. CCK-8 assay was conducted to measure cell viability. Data are expressed as the mean ± SEM. *P < 0.05, **P < 0.01, ***P < 0.001, vs. Ctrl; *n* = 3.

Considering the involvement of KLF4 in chemoresistance in cancer [29–32], we sought to investigate whether DUB3 regulates the response of HCC cells to chemotherapy through KLF4. We found that overexpression of DUB3 in Hep3B and HepG2 cells considerably enhanced KLF4 protein expression (Fig. 5A) and resulted in lower IC50 values of doxorubicin hydrochloride and cisplatin, respectively, compared with control (Fig. 5B), suggesting that DUB3 enhances chemosensitivity of HCC cells possibly via KLF4. In contrast, knockdown of DUB3 in Hep3B and HepG2 cells significantly attenuated KLF4 protein expression and reduced their sensitivity to 5-fluorouracil (5-FU) or cisplatin treatment. Importantly, re-expression of KLF4 in DUB3-silenced HCC cells completely restored DUB3 expression and the sensitivity to 5-FU or cisplatin treatment (Fig. 5D, E–H).

### KLF4 promotes DUB3 transcription by binding to the DUB3 gene promoter

**Fig. 6:**
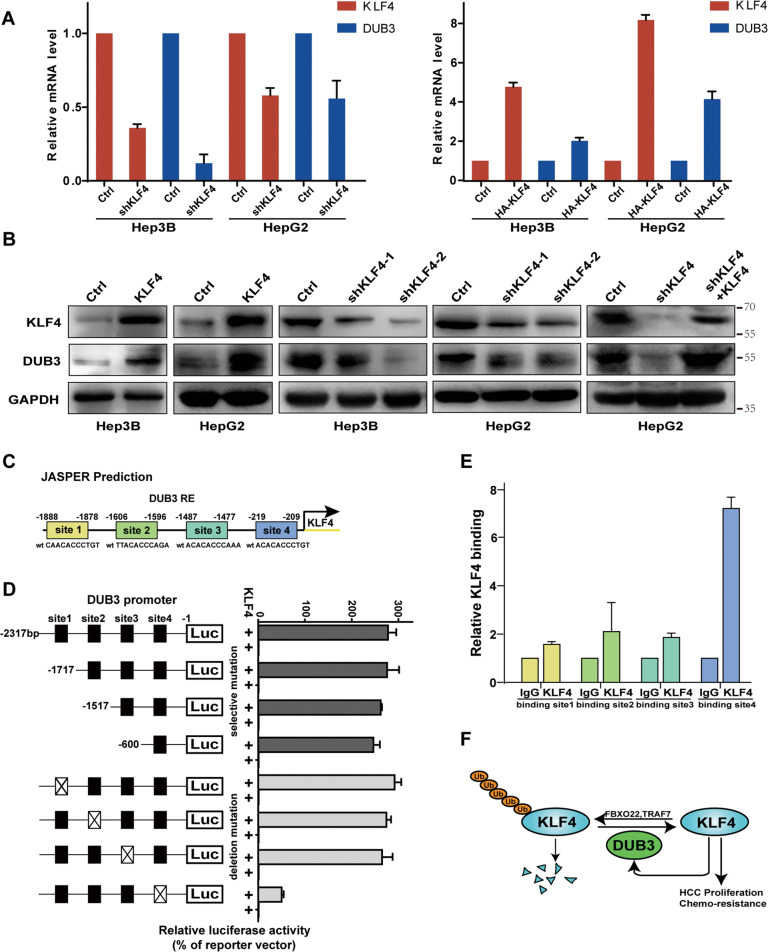
KLF4 promoted DUB3 transcription by binding to the DUB3 gene promoter. **A** Hep3B and HepG2 cells were stably transfected with vectors expressing shKLF4 or HA-KLF4. qRT-PCR was performed to measure the mRNA levels of DUB3 and KLF4. **B** Hep3B and HepG2 cells were stably transfected with vectors expressing shKLF4 and KLF4 alone or in combination. Western blot analysis was conducted to determine protein expression of DUB3 and KLF4. **C** The potential binding sites of KLF4 on DUB3 promoter were predicted using the JASPAR database (http://jaspar.genereg.net/). **D** 293T cells stably expressing control or KLF4 were transfected with the luciferase reporter plasmids containing the wild-type DUB3 promoter or DUB3 promoter with indicated deletion mutation (site 1, −1888 to −1878; site 2, −1606 to −1596; site 3, −1487 to −1477; site 4, −219 to −209). The relative luciferase activity was determined at 24 h after transfection. **E** ChIP assay was conducted using anti-IgG or anti-KLF4 antibody to identify the binding site of KLF4 on the DUB3 promoter in 293T cells. **F** A schematic diagram illustrates the DUB3/KLF4 positive feedback loop that inhibits tumor growth and overcomes chemoresistance in HCC.

In the Co-IP assay, we noticed that overexpression of KLF4 enhanced DUB3 protein expression in HEK293T cells (Fig. 2A), suggesting a positive feedback loop between DUB3 and KLF4. We further found that in Hep3B and HepG2 cells, overexpression of KLF4 significantly elevated the mRNA and protein levels of DUB3, whereas knockdown of KLF4 markedly reduced mRNA and protein levels of DUB3. Notably, re-expression of silenced KLF4 completely restored protein expression of DUB3 in HepG2 cells (Fig. 6A, B).

Since KLF4 is a transcription factor, we wondered whether KLF4 promotes DUB3 expression at the transcriptional level. Dual-luciferase reporter assay showed that KLF4 transactivated the promoter activity of the DUB3 gene (Supplementary Fig. 2A). Using the JASPER database, we predicted four potential KLF4 binding sites in the promoter region of the DUB3 gene (site 1, −1888 to −1878; site 2, −1606 to −1596; site 3, −1487 to −1477; site 4, −219 to −209; Fig. 6C). Dual-luciferase reporter assay revealed that deletion of site 4 dramatically inhibited the promoter activity of the DUB3 gene compared with deletion of other potential binding sites (Fig. 6D), suggesting that site 4 is essential for KLF4-induced DUB3 transcriptional activation. Moreover, the ChIP assay showed that only site 4 promoter fragment was enriched in the cell lysates immunoprecipitated by anti-KLF4 antibody (Fig. 6E), suggesting that KLF4 promotes DUB3 transcription by binding to the DUB3 gene promoter at −219 to −209 fragment.

### DUB3 expression positively correlates with KLF4 expression in HCC tissue and prognosis in HCC patients

**Fig. 7:**
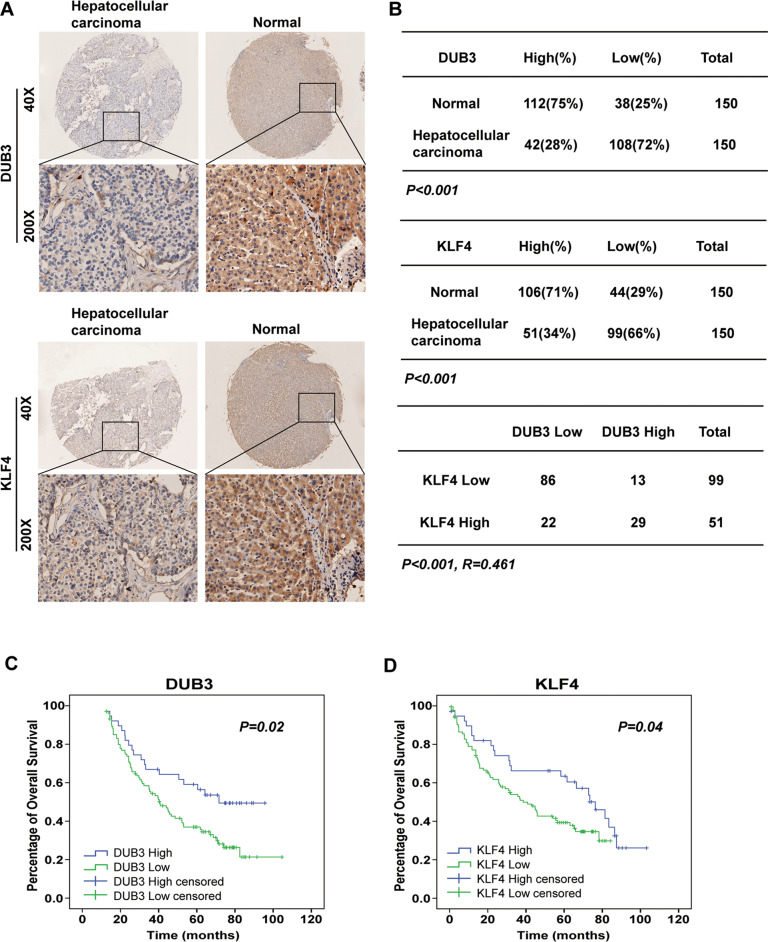
DUB3 expression positively correlated with KLF4 expression in HCC tissue samples and overall survival in HCC patients. **A** Immunohistochemical (IHC) staining was performed to detect KLF4 and DUB3 protein expression in paired HCC and paracancer tissue samples from 150 patients with HCC. Representative images are shown. Magnification 40×, 200×. **B** Quantification of DUB3 and KLF4 protein levels in HCC and paracancer tissue samples. The correlation between DUB3 and KLF4 protein expression in HCC samples was analyzed. **C**, **D** Kaplan–Meier survival analysis of HCC patients with low or high DUB3 and KLF4 protein levels based on IHC scoring.

Then, we examined the clinical significance of the DUB3/KLF4 loop in patients with HCC. As shown in Fig. 7A, B, IHC staining demonstrated that the percentage of HCC biopsies with low DUB3 protein expression was substantially higher than that of normal samples (72% vs. 25%, *P* < 0.001). A similar trend was observed in the percentages of HCC biopsies versus normal samples with low KLF4 protein expression (66% vs. 29%, *P* < 0.001; Fig. 7A, B). A strong positive correlation was observed between DUB3 and KLF4 protein expression in HCC biopsies (*P* < 0.001, *R* = 0.461; Fig. 7B). Besides, Kaplan–Meier curve analysis showed that HCC patients in the low DUB3/KLF4 expression group had notably poorer overall survival compared with those in the high DUB3/KLF4 expression group (Fig. 7C, D). Moreover, multivariate analysis identified DUB3 expression as an independent predictor for overall survival and cumulative recurrence (Table 1).

**Table 1:** Univariate and multivariate analyses of factors associated with survival and recurrence in 150 HCC.

Factors	Overall survival			Cumulative recurrence		
	Univariate	Multivariate	*p-*value	Univariate	Multivariate	*p-*value
	*p-*value	HR, 95%CI		*p-*value	HR, 95%CI	
Sex (female vs. male)	0.75		NA	0.436		NA
Age(y) (>50 vs. ≤50)	0.896		NA	0.598		NA
HBsAg (positive vs. negative)	0.976		NA	0.537		NA
Liver cirrhosis (yes vs. no)	0.808		NA	0.218		NA
Serum AFP, ng/mL (>20 vs. ≤20)	0.191		NA	0.1		NA
Tumor size, cm (>5 vs. ≤5)	<0.001		NS	<0.001		NS
Tumor number (multiple vs. single)	0.225		NA	0.984		NA
Tumor differentiation (III/IV vs. I/II)	0.346		NA	0.099		NA
Vascular invasion (yes vs. no)	0.001		NS	0.032		NS
TNM stage (III/IV vs. I/II)	<0.001	1.908, 1.199–3.03	0.006	<0.001	2.012, 1.265–3.19	0.003
BCLC stage (B/C vs. A/0)	<0.001	2.423, 1.333–4.40	0.004	<0.001	2.152, 1.214–3.81	0.009
DUB3 expression (low vs. high)	0.037	1.951, 1.151–3.30	0.013	0.034	1.854, 1.097–3.13	0.021

*HR* Hazard ratio, *95%CI* 95% confidence interval, *NA* not adopted, *NS* not significant, *HBsAg* hepatitis B surface antigen, *AFP* alpha-fetoprotein, *TNM* T, tumor, N, node, M, metastasis, *BCLC* Barcelona clinic liver cancer, *Cox* proportional hazards regression model.

## Discussion

KLF4 acts as an oncogene or tumor suppressor depending on the types of cancer [10–14], indicating the necessity of a better understanding of its cellular context in HCC. We have previously demonstrated that KLF4 acts as a tumor suppressor in HCC by upregulating the MET pathway [15]. The ubiquitination and degradation of KLF4 play important roles in HCC progression [16, 17]. Xin et al. have reported that the deubiquitinase USP10 regulates KLF4 stability and suppresses lung tumorigenesis [33]. Zou et al. have reported that ATXN3 promotes breast cancer metastasis by deubiquitinating KLF4 [34]. However, we did not find significant changes in KLF4 expression in ATXN3-overexpressing Hep3B cells (Supplementary Fig. 1C), suggesting that KLF4 is regulated by other DUBs in HCC cells. In this study, after screening a panel of deubiquitination enzymes (DUBs), we found that DUB3 deubiquitinated and stabilized KLF4 in HCC cells. Knockdown of DUB3 promoted cell proliferation and induced chemoresistance in HCC cells via suppressing KLF4 expression. On the other hand, KLF4 upregulated DUB3 transcription by binding to the promotor of DUB3, suggesting a positive feedback loop of DUB3/KLF4 in HCC cells. Together with the positive correlations of DUB3 with KLF4 in human HCC tissue samples and overall survival in HCC patients, our findings suggest that activating the DUB3/KLF4 loop may prevent tumor growth and chemoresistance in HCC.

The role of DUB3 in cancer remains controversial. Previous studies, including ours, have shown that DUB3 is a bona fide Snail1 DUB that promotes tumor cell invasion, migration, and metastasis in breast cancer [20]. We also have found that CDK4/6 inhibitor prevents breast cancer metastasis by targeting the CDK4/6-DUB3-Snail axis [20]. On the other hand, DUB3 regulates a correct DNA damage response by controlling H2AX ubiquitination [19] and is involved in cell cycle progression through stabilizing cyclin A and cdc25A [23, 24]. Nguyen et al. have reported that DUB3 acts as a tumor suppressor by limiting YAP activity [35]. In this study, we identified that DUB3 upregulated KLF4 expression through deubiquitinating and stabilizing KLF4 in HCC cells. Consistent with our previous finding showing that KLF4 suppresses liver cancer growth [15], the results of this study showed that DUB3 inhibited HCC cell proliferation in vitro and tumor growth in vivo in a KLF4-dependent manner. Because DUB3 has been demonstrated to drive chemoresistance in colorectal cancer and ovarian cancer via stabilizing NRF2 and MCL1 [31, 36], we explored whether DUB3 affects the response of HCC cells to the commonly used chemotherapeutic drugs for HCC treatment. Our results showed that overexpression of DUB3 enhanced the sensitivity of HCC cells to doxorubicin hydrochloride and cisplatin, whereas knockdown of DUB3 reduced the sensitivity of HCC cells to 5-FU and cisplatin. Importantly, overexpression of KLF4 restored the chemosensitivity suppressed by DUB3 silencing. These findings suggest that DUB3 overcomes chemoresistance in HCC through KLF4.

Some DUBs acquire their substrates by binding the target protein and cleaving the Ub chain from the target [37, 38]. Our Co-IP assay revealed physical interactions between endogenous DUB3 and KLF4 in HEK293T cells and identified that 117–181 and 181–389 residues of KLF4 were both essential for the binding of DUB3. In the results of the Co-IP assay, we noticed that overexpression of KLF4 enhanced DUB3 protein expression in HEK293T cells, suggesting a reciprocal relationship between KLF4 and DUB3. Given that KLF4 is an important transcription factor, we investigated the possible binding sites of KLF4 in the promoter region of DUB3 and identified that KLF4 activated DUB3 transcription by binding to the −209 to −219 fragment on the DUB3 promoter. To the best of our knowledge, this is the first report describing the positive feedback loop between DUB3 and KLF4. Our in vitro and in vivo data suggest that overexpression of DUB3 and/or KLF4 may activate the DUB3/KLF4 loop to prevent tumor growth and chemoresistance, whereas knockdown of DUB3 and/or KLF4 may block the DUB3/KLF4 loop to facilitate tumor growth and chemoresistance in HCC. Future studies, such as establishing the DUB3 or KLF4 knockout models, are required to further explore the role of the DUB3/KLF4 loop in the pathogenesis of HCC.

The DUB3/KLF4 loop also exhibited clinical significance in patients with HCC. In the tissue microarray containing 150 pairs of HCC tissue and adjacent normal tissue samples, we observed significantly decreased DUB3 and KLF4 protein levels in HCC tissue samples compared with those in normal controls. We also observed a strong positive correlation between DUB3 and KLF4 protein expression in HCC biopsies. Besides, lower expression of DUB3 or KLF4 correlated with poorer overall survival in HCC patients. Moreover, low DUB3 expression predicted shorter overall survival and cumulative recurrence in HCC patients. These data are consistent with what we observed in HCC cells and xenograft mouse model, suggesting the feasibility of incorporating our bench-top research into clinical practice.

## Materials and methods

### Animals

All animal care and experimental procedures were approved by the Ethical Committee of Shanghai East Hospital (approval number: SCXK2017-0005). All animal experiments were performed according to the Guidelines for Proper Conduct of Animal Experiments at Shanghai East Hospital. BALB/c male nude mice (5-week old) were purchased from Shanghai SLAC Laboratory Animal Co., Ltd. and maintained under specific pathogen-free conditions. Mice were randomly divided into four groups (*n* = 4/group). Each mouse was subcutaneously injected with a 200 µL mixture containing 100 µL matrix gel, 100 µL 1× phosphate-buffered saline (PBS), and 2 × 10^6^ Hep3B cells stably expressing small hairpin RNA against DUB3 (shDUB3), shKLF4, shDUB3 + shKLF4, or control shRNA. Tumor volumes were monitored for 4 weeks and calculated as height × width × length × 1/2. At the end of the study, the tumors were collected and weighted.

### Cell lines and cell culture

The HEK293T cell line, human HCC cell lines HepG2 and Hep3B, and normal liver cell line LO2 were purchased from the American Type Culture Collection (ATCC; Manassas, VA, USA) and grown in Dulbecco’s modified Eagle’s medium (Corning Inc., Corning, NY, USA) supplemented with 10% fetal bovine serum (Gibco, New York, NY, USA) and 1% penicillin-streptomycin (Gibco) at 37 °C in a humidified atmosphere of 5% CO_2_.

### Overexpression and knockdown

Hemagglutinin (HA)-tagged-KLF4 plasmids were purchased from Asia-Vector Biotechnology (Shanghai, China). KLF4 deletion mutant was constructed as follows: site 1, 1 to 117aa; site 2, 117 to 181aa; site 3, 181 to 389aa; site 4, 389 to 513aa.Flag-tagged-DUB3 was obtained from GenePharma (Shanghai, China). DUB3 C89S mutant was kindly provided by Jian Yuan [27]. The shRNA sequences were as follows: DUB3 shRNA-1: 5′-GCAGGAAGATGCCCATGAATT-3′; DUB3 shRNA-2: 5′-CACAAGCAGGTAGATCATCAC-3′; KLF4 shRNA-1: 5′-GCTCCATTACCAAGAGCTCAT-3′; KLF4 shRNA-2: 5′-GCCAGAATTGGACCCGGTGTA-3′. Cells were transfected with the plasmids using lipo2000 following the manufacturer’s instructions.

### Quantitative real-time PCR (qRT-PCR)

Total RNA was isolated using Trizol reagent (Takara Biotechnology) according to the manufacturer’s instructions. cDNA was synthesized using TaqMan RT Reagents Kit (Applied Biosystems). PCR was performed using a QuantiTect SYBR Green PCR Kit (Takara) on an Applied Biosystems 7500 System. GAPDH was used as an internal reference. PCR reactions were performed in triplicate. Gene expression was quantified using the 2^−ΔΔct^ method. The primer sequences were summarized in Supplementary Table 1.

### Western blot analysis

Cells were lysed with RIPA buffer on ice, followed by centrifugation at 12,000 rpm for 5 min. Protein concentrations were measured using a BCA Protein Assay Reagent Kit (Beyotime, Shanghai, China). Proteins (30 µg) were separated on a 10% SDS gel and transferred to a polyvinylidene fluoride membrane, followed by 1 h of blocking with 5% skim milk. The membrane was then incubated with anti-DUB3 (ab129931; Abcam, Cambridge, UK), anti-KLF4 (11880-1-AP; Proteintech, Rosemont, IL, USA or Ab106629; Abcam), anti-Ub (Cell Signaling Technology, Danvers, MA, USA), anti-Flag M2 (F1804; Sigma-Aldrich, St. Louis, MO, USA), anti-HA (H9658; Sigma-Aldrich), or anti-GAPDH (10494-1-AP; Proteintech, Rosemont, IL, USA) antibody overnight at 4 °C, followed by three washes with Tris-buffered saline containing 0.1% Tween 20 (TBST). The membrane was then incubated with horseradish peroxidase-conjugated secondary antibody (Cell Signal Technology, Beverly, MA, USA) for 1 h at room temperature. After additional three washes with TBST, the protein bands were visualized using an enhanced chemiluminescence system and analyzed using the ImageJ software (US National Institutes of Health).

### Cell proliferation assay

HCC cell proliferation was analyzed using a cell counting kit-8 (CCK-8; Dojindo) assay. A total of 2 × 10^3^ Hep3B or HepG2 cells were seeded in 96-well plates (Corning) and incubated overnight at 37 °C in 5% CO_2_. After transfection or drug treatment, cells were incubated with 10 µL CCK-8 at 37 °C for 1.5 h. The optical density at 450 nm was measured using a multi-well plate reader (Thermo Fisher Multiskan FC, USA). Each experiment was repeated three times.

### Colony formation assay

In order to assess colony formation, cells were plated in a six-well plate (5 × 10^2^ cell/well) and cultured in DMEM supplemented with 10% FBS and 1% penicillin-streptomycin for 2 weeks. The colonies were fixed with methanol and stained with 0.1% crystal violet and quantified the colony number with an Olympus microscope.

### Cycloheximide chase assay

Cells were harvested at different time points after cycloheximide treatment. Cells lysed with NETN were collected for Western blot analysis.

### Intracellular and extracellular deubiquitination assays

For intracellular deubiquitination assay, HEK293T cells were transfected with wild-type or mutant FLAG-DUB3, HA-KLF4, or His-Ub, followed by MG132 (25 μM) treated for 5 h. Cells were lysed in 120 μL NTEN buffer (pH 6.8, 62.5 mM Tris-HCl, 10% glycerol, 2% SDS, 20 mM NEM, and 1 mM iodoacetamide) and boiled for 15 min. The cell lysates were immunoprecipitated with FLAG/HA beads at 4 °C for 4 h, followed by Western blot analysis.

For the extracellular deubiquitination assay, His-Ub-conjugated HA-KLF4 was isolated and purified from HEK293T cells as previously described [36, 39]. The purified wild-type and mutant DUB3 were incubated with Ub-HA-KLF4 in HA lysis buffer. The proteins were eluted using HA-peptides (Sigma-Aldrich), followed by Western blot analysis.

### Dual-luciferase reporter assay

DUB3 promoter fragments were cloned into the GV-354 vector (Genechem, Shanghai, China). The KLF4-overexpressing or control cells were transfected with GV-354-DUB3-promotor reporter plasmids. At 48 h after transfection, a dual-luciferase reporter assay was conducted using the dual-luciferase reporter assay system (E1910; Promega, Madison, WI, USA) according to the manufacturer’s protocol. The luciferase activity was measured using GloMax 96 microplate luminometer (Promega). The firefly luciferase enzyme activity was normalized to the Renilla luciferase enzyme activity.

### Co-immunoprecipitation (Co-IP) assay

Cells were harvested and washed twice with ice-cold PBS. Following lysis with RIPA buffer and centrifugation, the supernatant was incubated with corresponding antibodies and protein A/G-agarose beads (Amersham Biosciences, Little Chalfont, UK) at 4 °C for 4 h. The immunoprecipitates were collected by centrifugation at 1000 × *g* for 5 min at 4 °C. The pellet was resuspended in RIPA buffer containing 1× loading buffer, followed by Western blot analysis.

### Chromatin Immunoprecipitation (ChIP) assay

The ChIP assay was performed as previously described [40]. The anti-KLF4 antibody (Abcam, Cambridge, UK) was used as a specific antibody for ChIP assay.

### Immunohistochemical (IHC) staining

Tissue microarrays containing 150 pairs of HCC tissue and adjacent normal tissue samples were kindly provided by Dr. Yu (Shanghai Longhua Hospital). DUB3 and KLF4 expression were detected by anti-DUB3 (dilution 1:100) and anti-KLF4 (dilution 1:200) antibodies using a HRP/DAB kit (#DAB50; MilliporeSigma, Burlington, MA, USA). The results of IHC were blindly assessed by experienced pathologists. The IHC scoring was performed as previously described [41]. The patients were divided into low and high DUB3/KLF4 expression groups according to the mean expression level of DUB3/KLF4.

### Statistical analysis

Data were expressed as the mean ± standard deviation (SD) in cell survival assay. Data were expressed as the mean ± SD in the tumor xenograft assay. Data were expressed as the mean ± standard error of the mean from three independent experiments in cell proliferation assays. Statistical analysis was performed using SPSS 23.0 software (IBM, Armonk, NY, USA). The Kaplan–Meier analysis was used to analyze the correlation of DUB3 or KLF4 expression with the overall survival of HCC patients. Differences between the groups were assessed using analysis of variance followed by the Student’s *t*-test and *χ*^2^ test. A *P* value less than 0.05 was considered statistically significant.

## Supplementary information

Supplementary Materials

western blot original data

Manuscript Related File

## Data Availability

Data sharing is not applicable to this article as no datasets were generated or analysed during the current study.
