# Supplementary material for: DUB3/KLF4 combats tumor growth and chemoresistance in hepatocellular carcinoma
Source: Cell Death Discov. 2022 Apr 5;8:166. doi: 10.1038/s41420-022-00988-5 (PMC8983766; doi:10.1038/s41420-022-00988-5)
Supplement: Supplementary file 1 — Supplementary Materials [file 41420_2022_988_MOESM1_ESM.pdf]

Supplementary Materials

Supplementary Figure 1. A HEK293T cells transfected with indicated deubiquitinases were subjected to Western blotting (Fig.1A), and the chart shows the KIF4 protein level relative to GAPDH. **B** Hep3B cells were stably transfected with shDUB3 or control shRNAs. qRT-PCR was performed to measure mRNA levels of DUB3 and KLF4. **C** Hep3B cells were transfected with HA-ATXN3. Western blot analysis was performed to detect HA and KLF4 protein expression. **D** Western blot analysis was performed to detect DUB3 and KLF4 expression in Hep3B cells stably expressing shDUB3, shKLF4, or shDUB3 + shKLF4. **E** HEK293T and HepG2 cells were transfected with shDUB3. Western blot analysis was performed to detect DUB3 and KLF4 expression.

Supplementary Figure 2. A The DUB3 promoter luciferase construct (-2317–-1) was cotransfected with or without KLF4 into Hep3B cells. The promoter activities were detected using the dual-luciferase reporter assay system. **B** Agarose gel electrophoresis was performed to confirm the sizes of chromatin fragments in ChIP assay.

Supplementary Table 1. Primer sequences for quantitative real-time PCR.

Supplementary Fig.1.

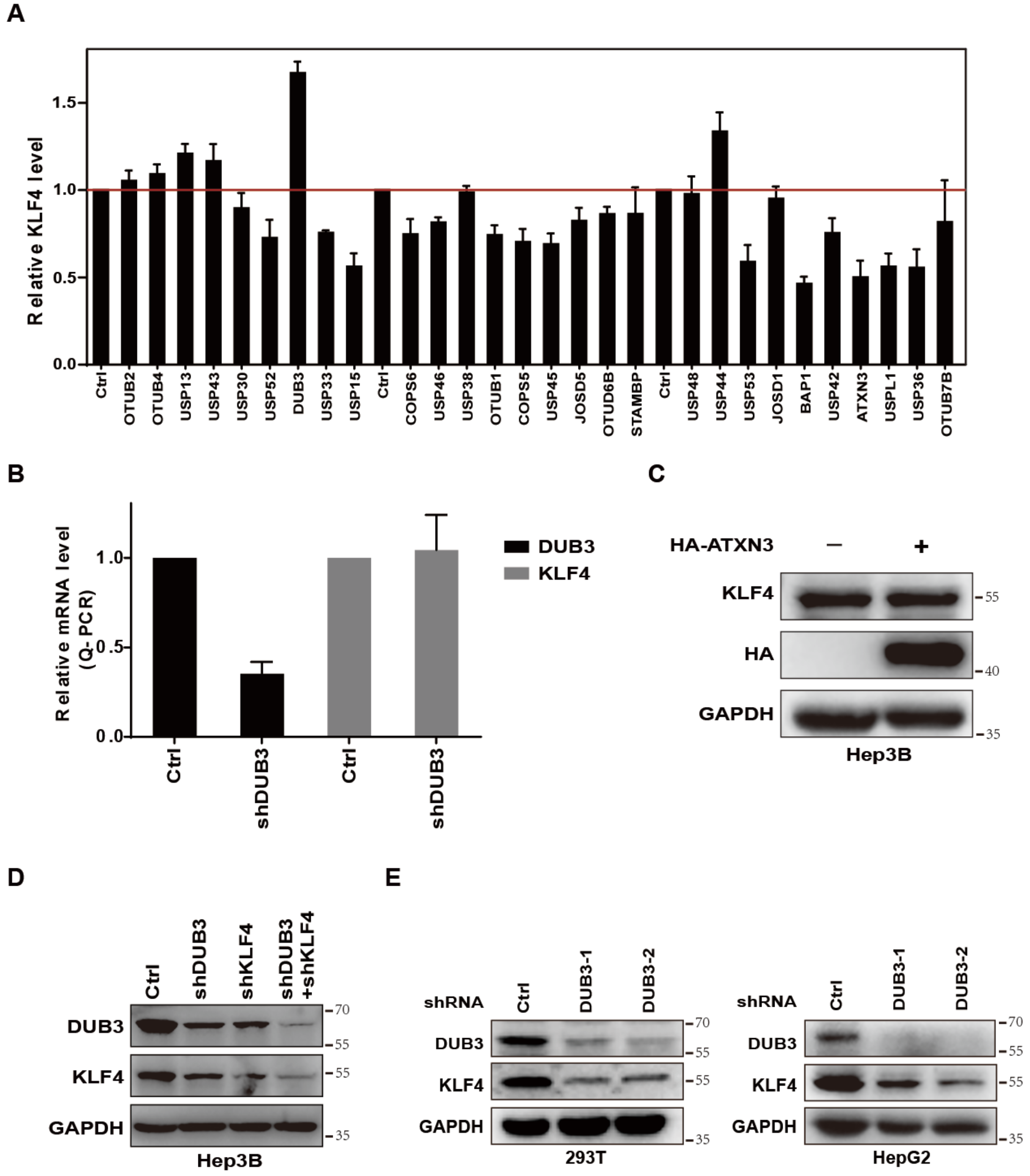

Supplementary Fig.2.

A

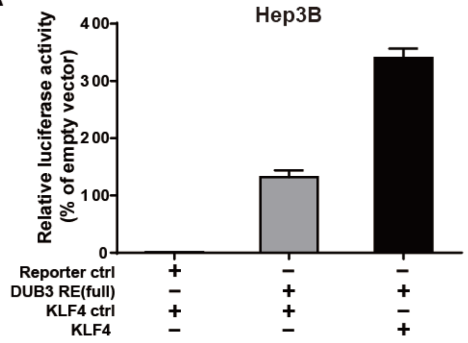

B

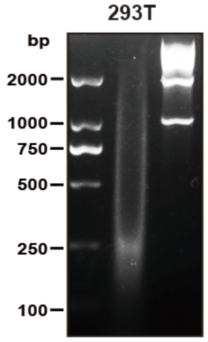

Supplementary Table 1. Primer sequences for quantitative real-time PCR

Genes	Forward primer (5'–3')	Reverse primer (5'–3')
DUB3	TCCTCTATGCTGTGCTGGTCCAC	GTTGCTCGCCTGTCTGTGTCTTC
KLF4	ACCCTGGGTCTTGAGGAAGT	CATGAGCTCTTGGTAATGGAGC
GAPDH	AGCCACATCGCTCAGACAC	GCCCAATACGACCAAATCC
P27	AAACGTGCGAGTGTCTAACG	CTCTGCAGTGCTTCTCCAAG
P57	CGATGGAGCGTCTTGTCG	CGTAATCCCAGCGGTTCTG
