# Supplementary material for: DUB3/KLF4 combats tumor growth and chemoresistance in hepatocellular carcinoma
Source: Cell Death Discov. 2022 Apr 5;8:166. doi: 10.1038/s41420-022-00988-5 (PMC8983766; doi:10.1038/s41420-022-00988-5)

Figure 1.A

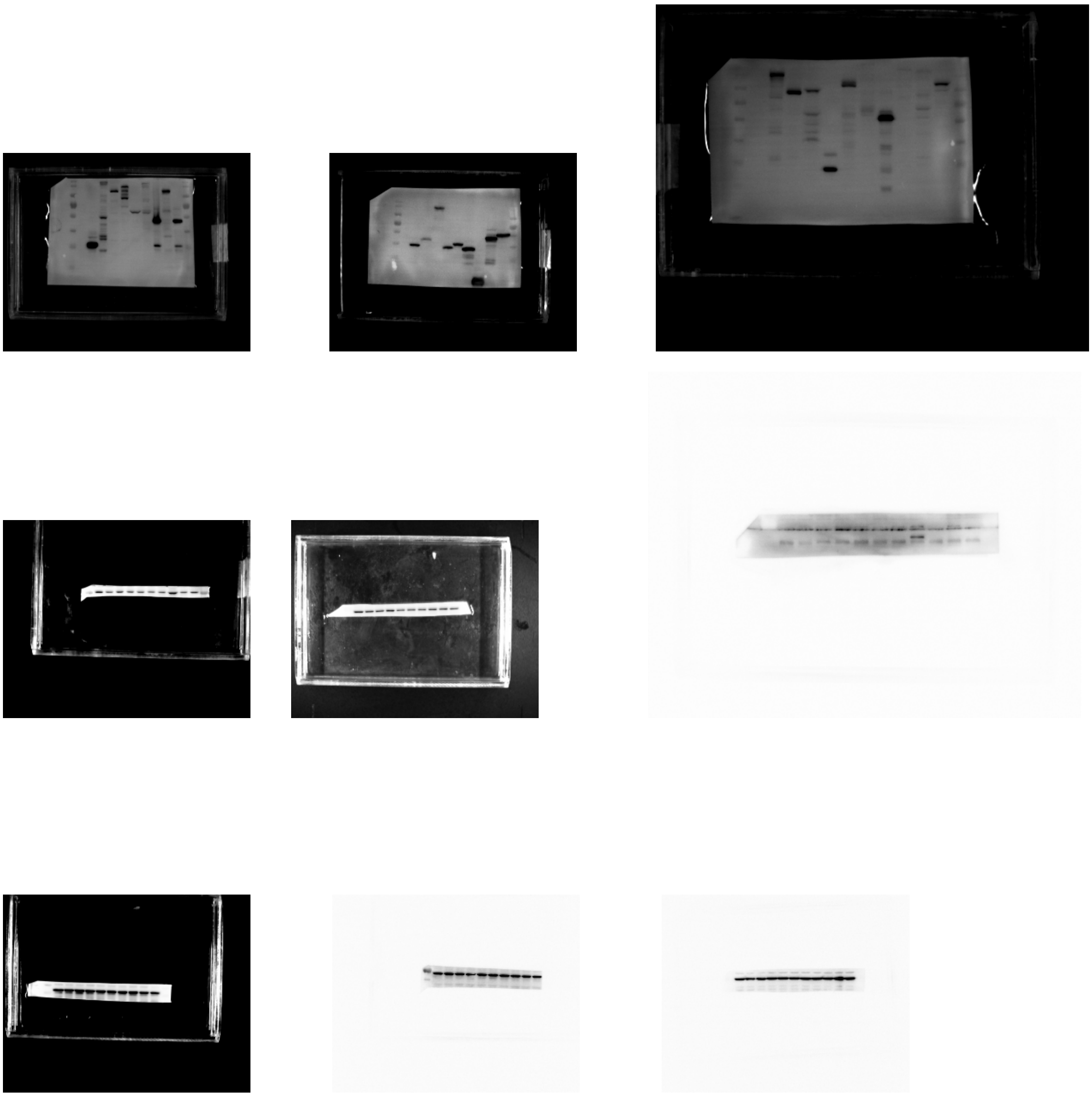

Figure 1.B

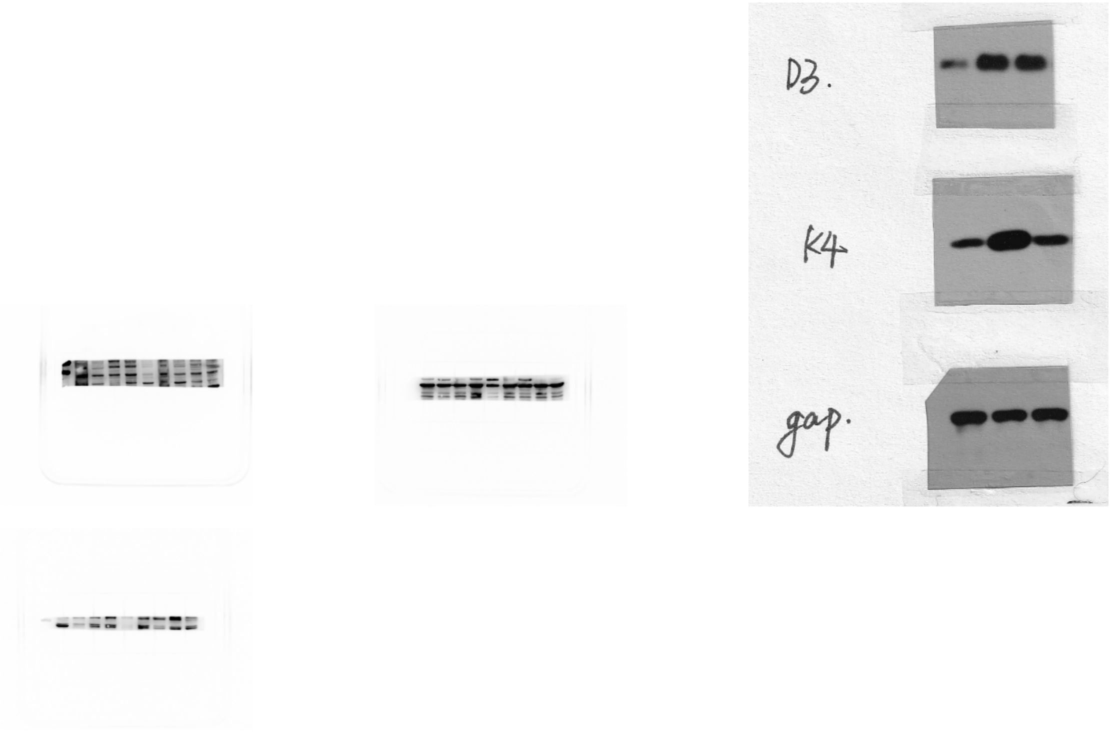

Figure 1.C

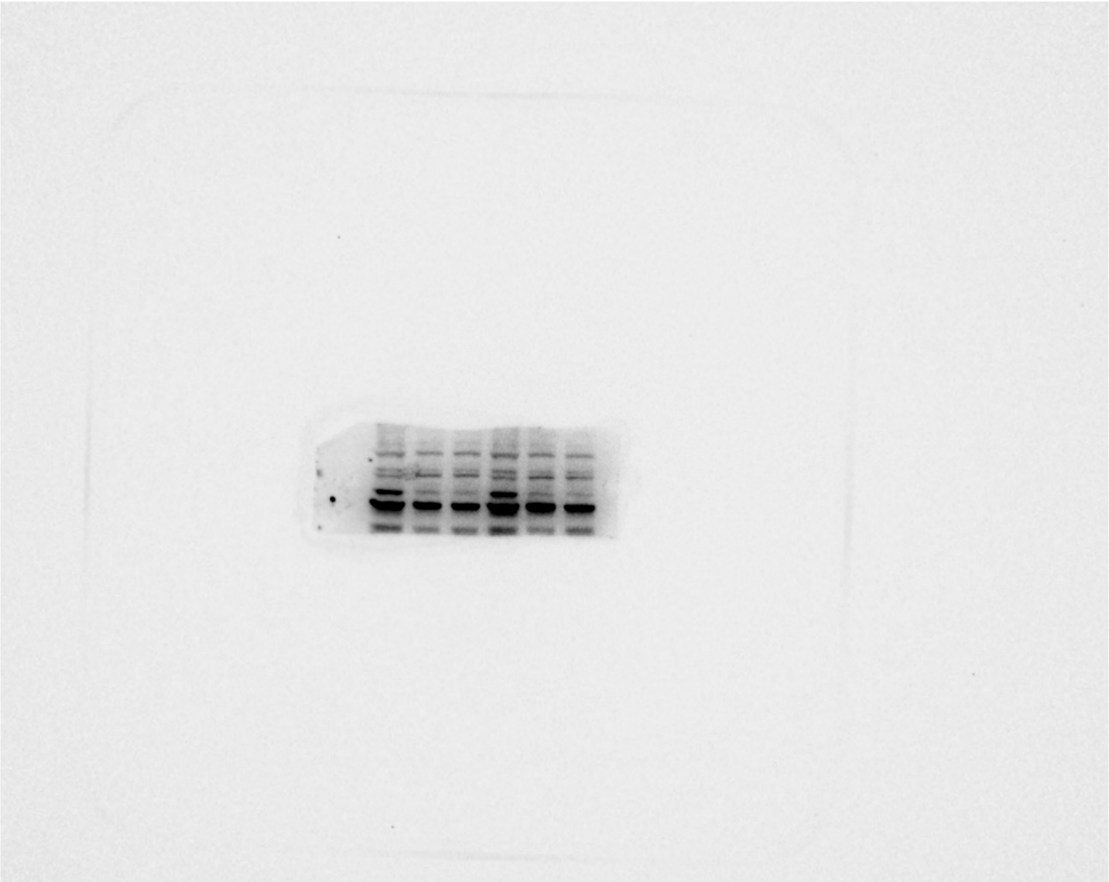

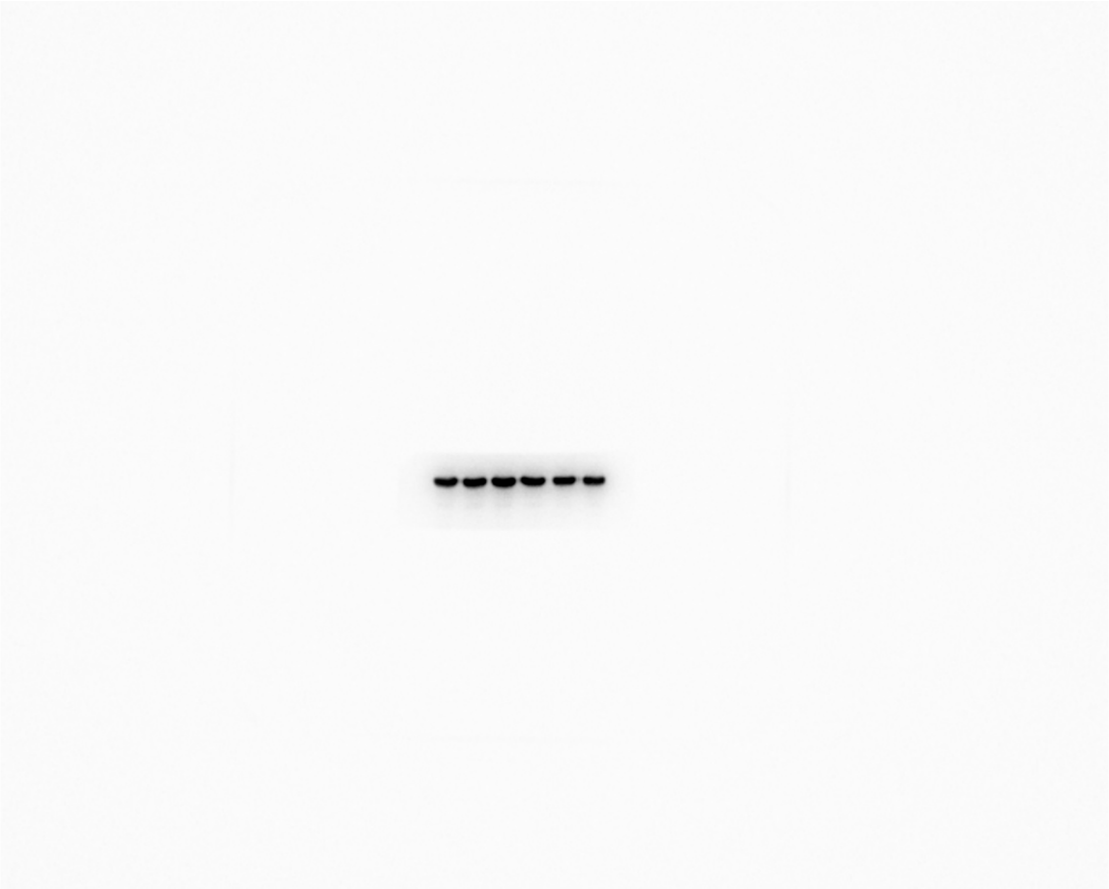
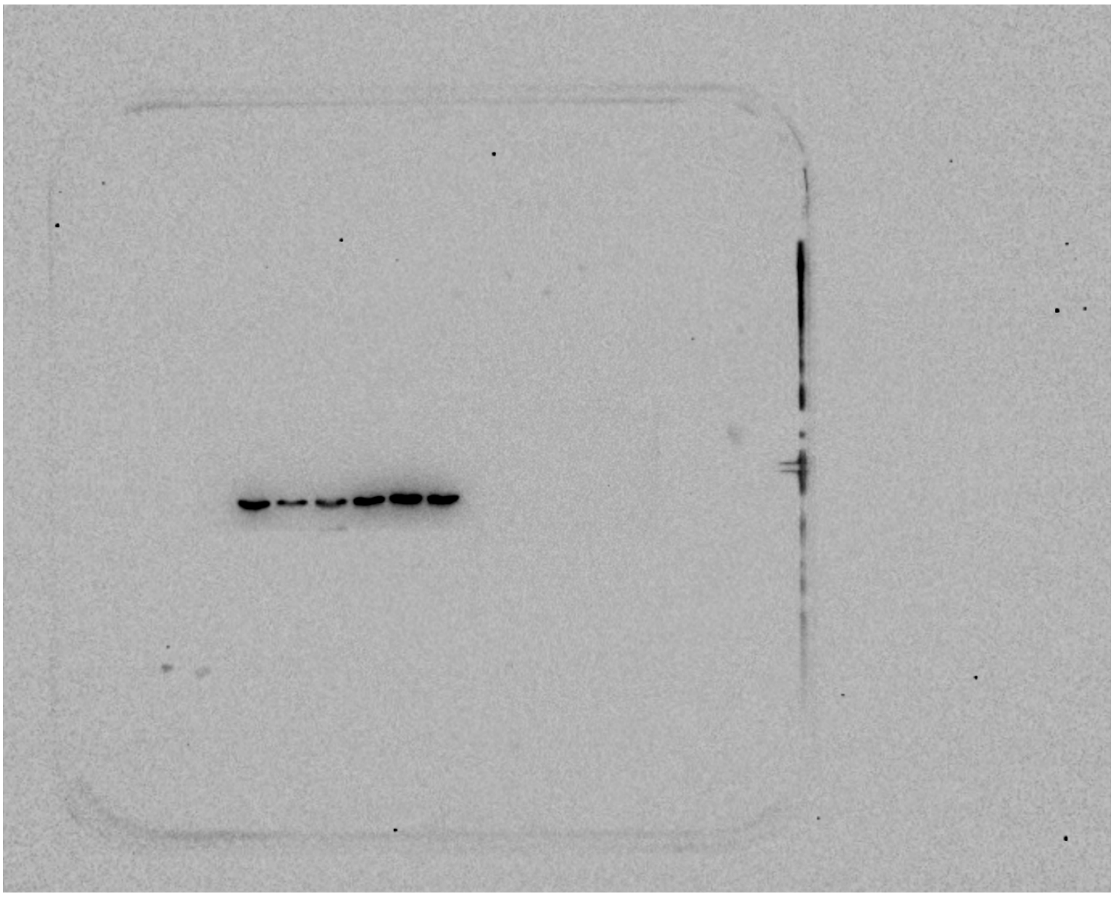

Figure 1.E

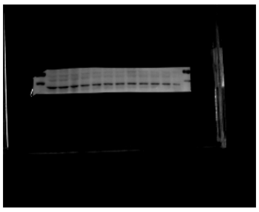

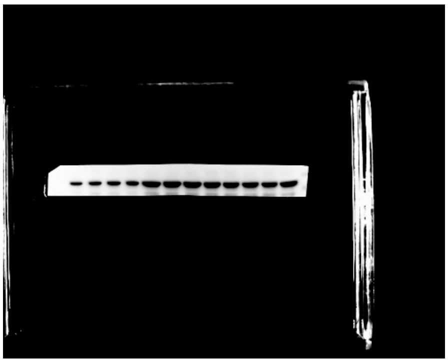

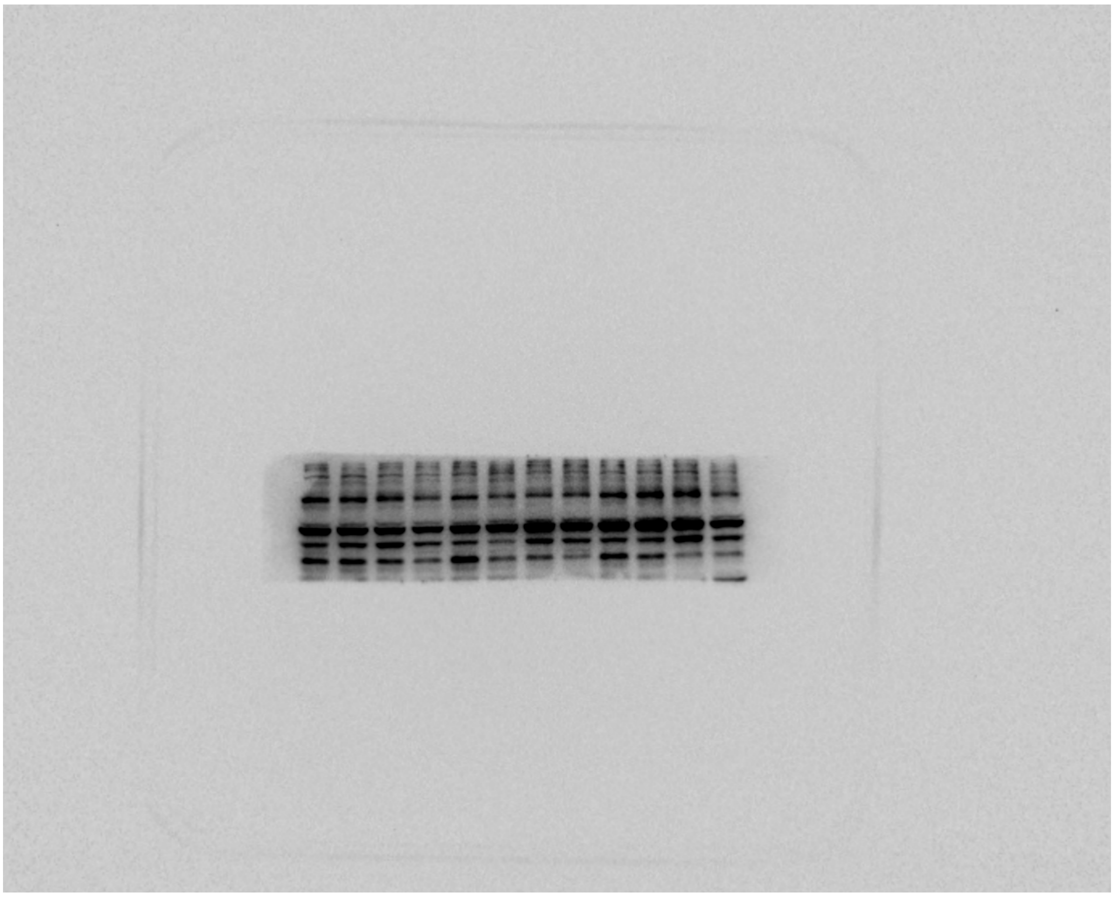

Figure 2.A,B

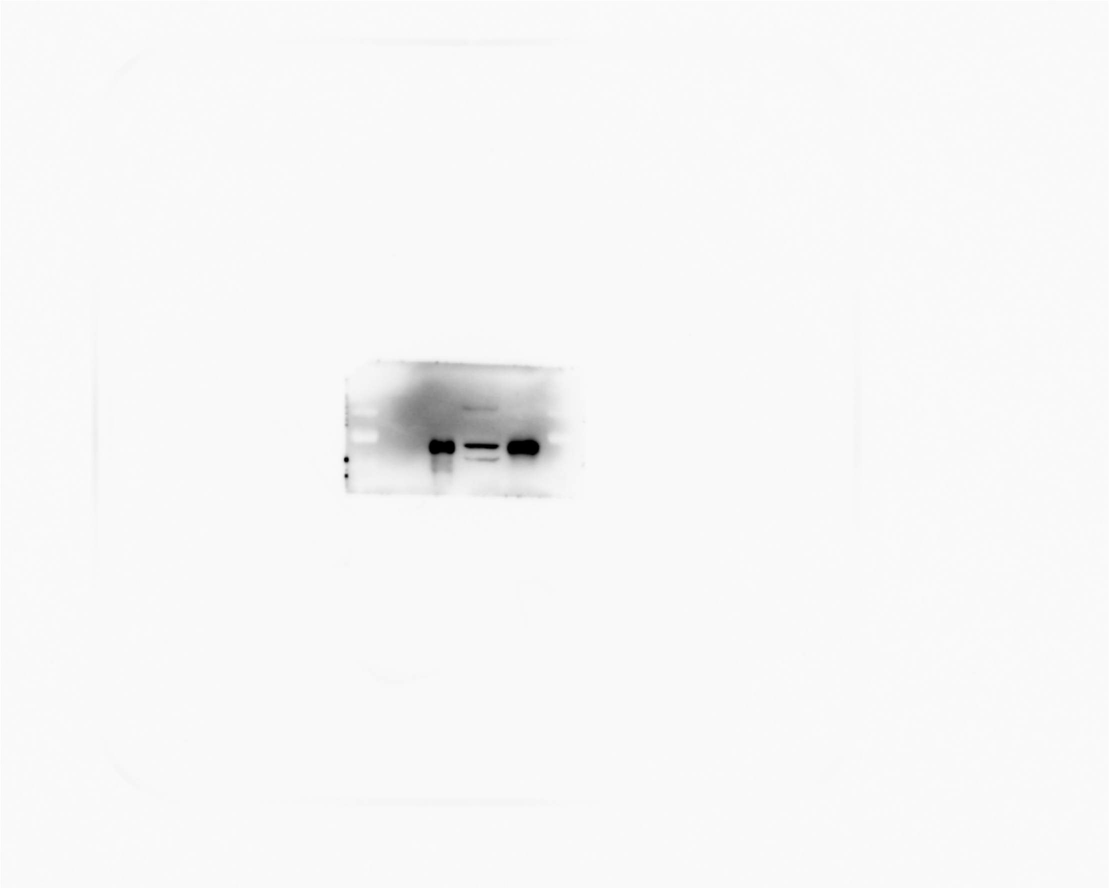

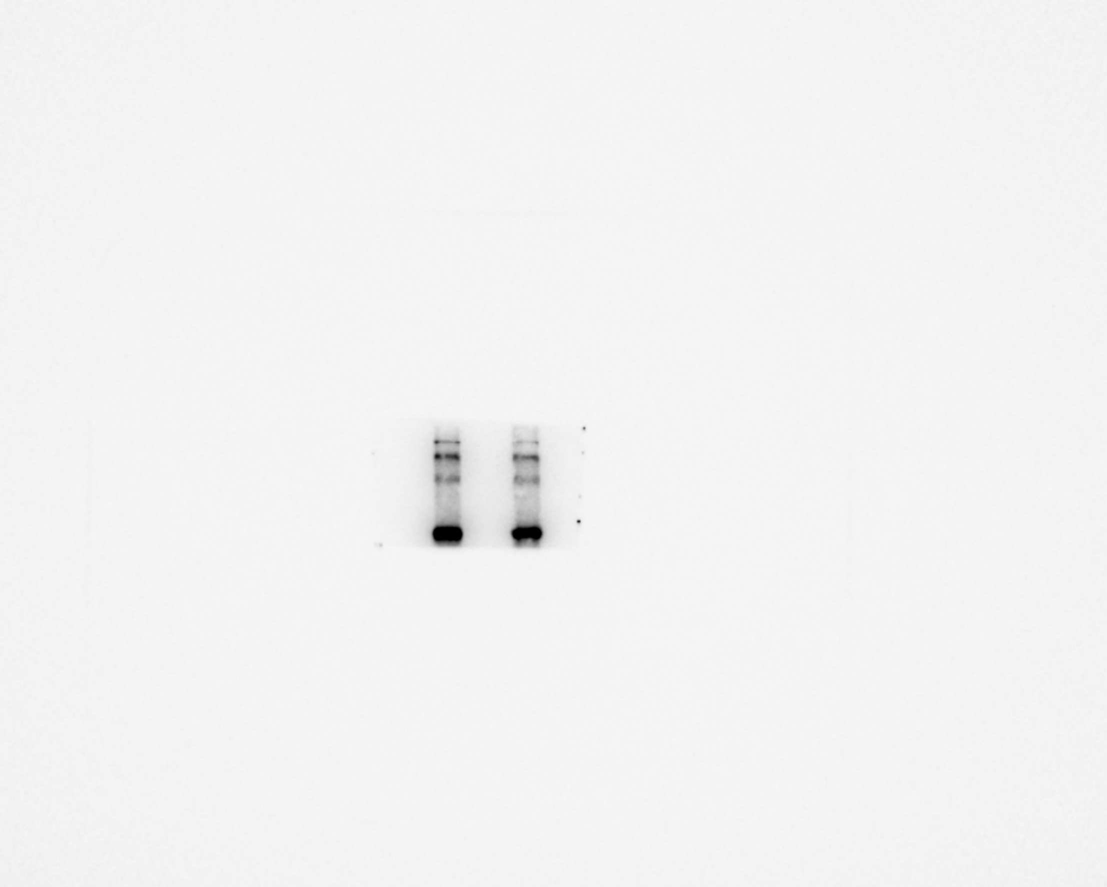

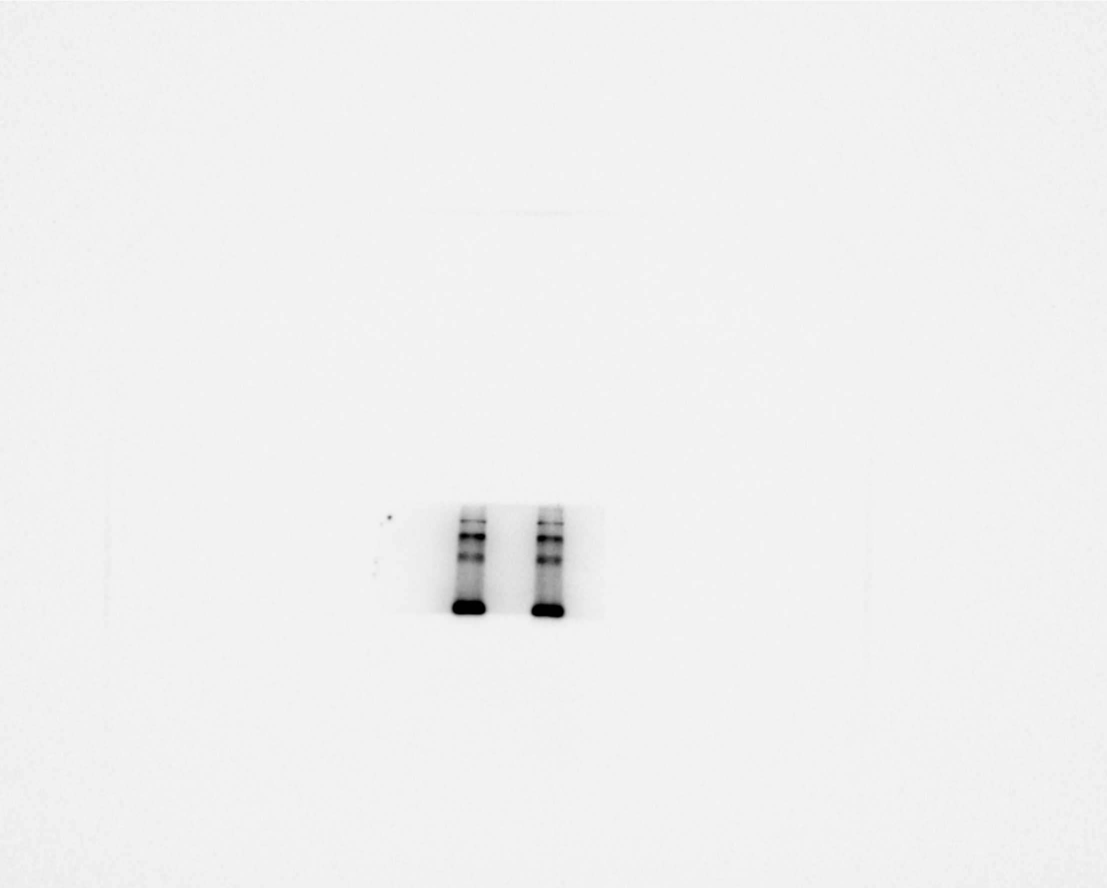

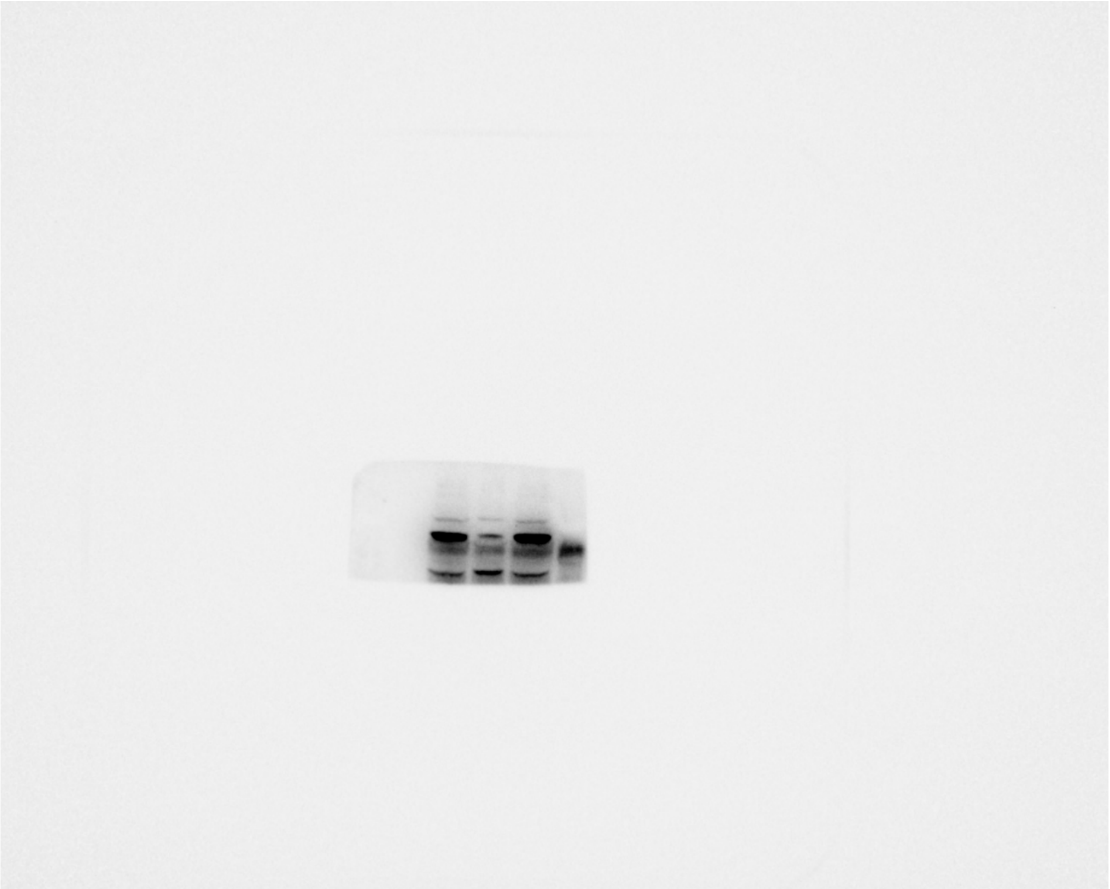

Figure 2.C,D

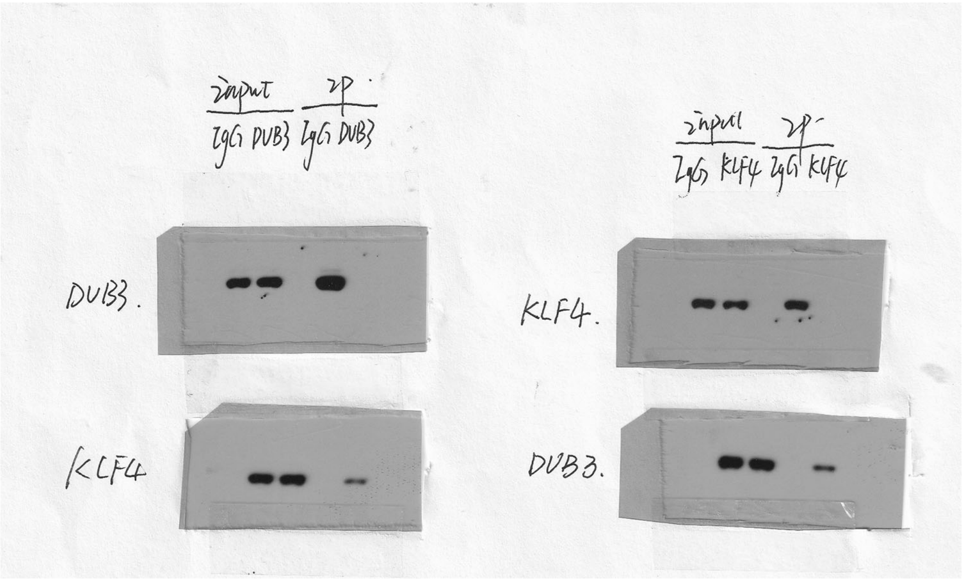
input IP
IgG DUB3 IgG DUB3
DUB3.
KLF4
input IP
IgG KLF4 IgG KLF4
KLF4.
DUB3.

Figure 2.F

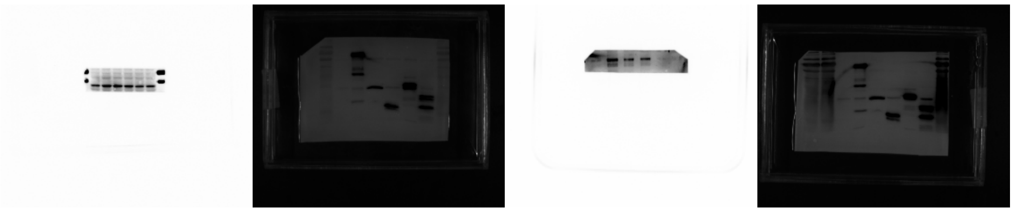

Figure 3.A

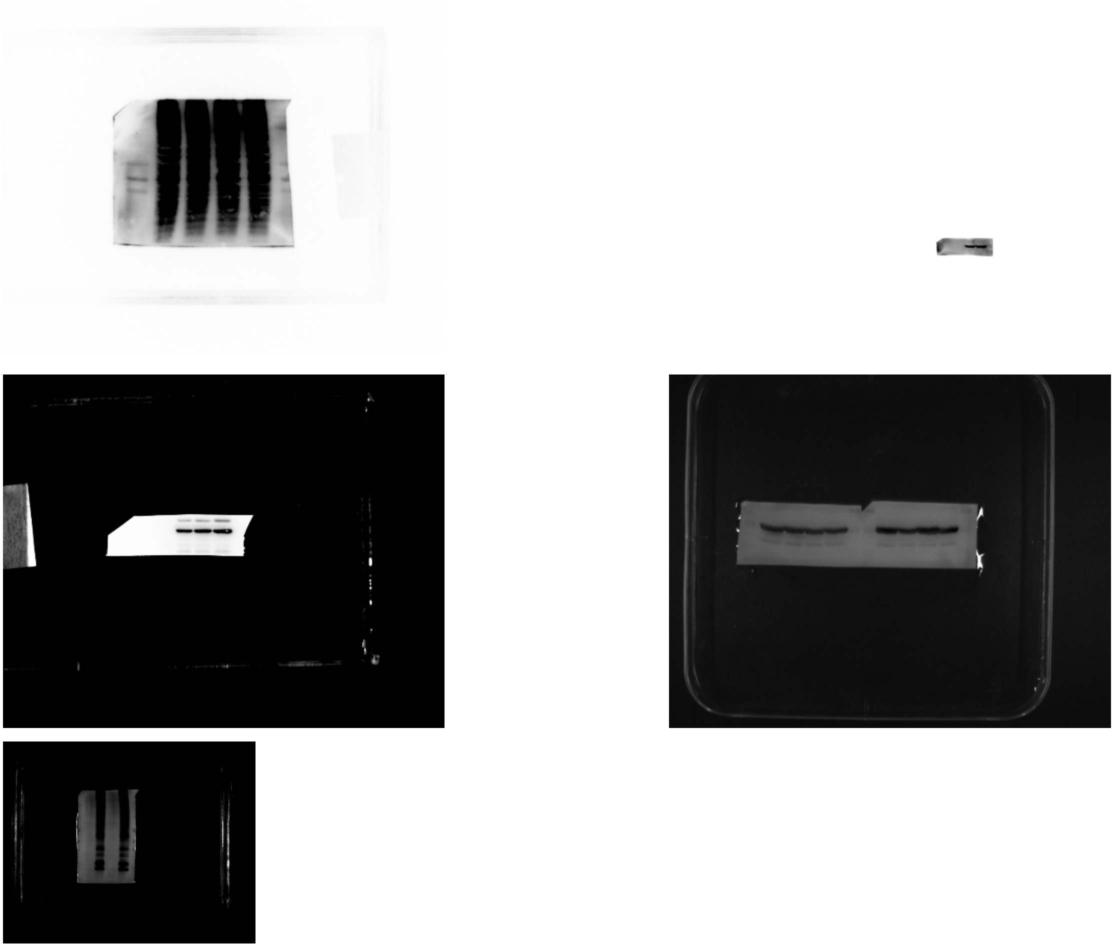

Figure 3.B

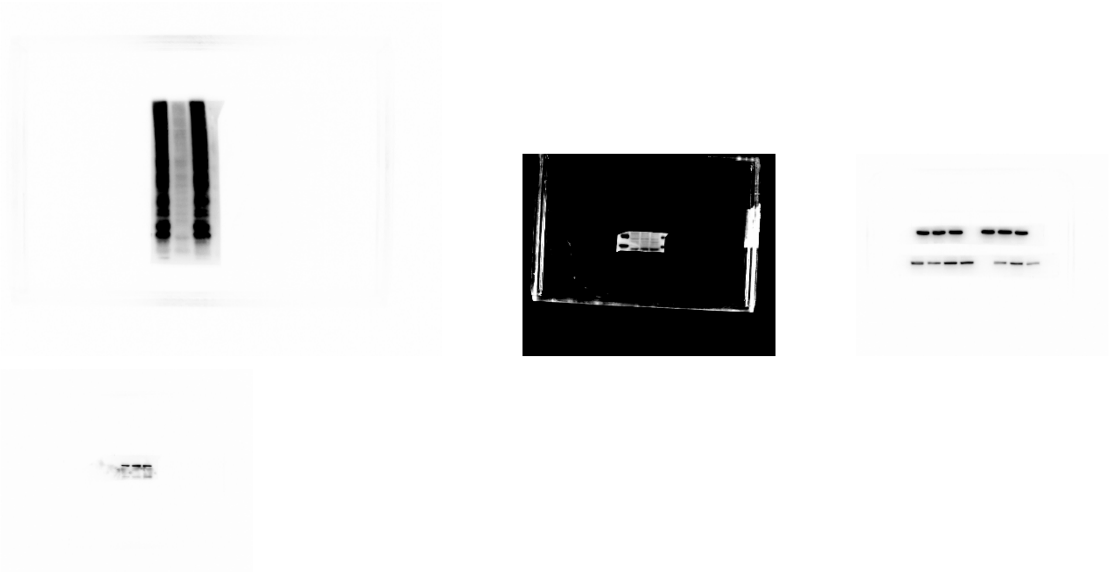

Figure 3.C

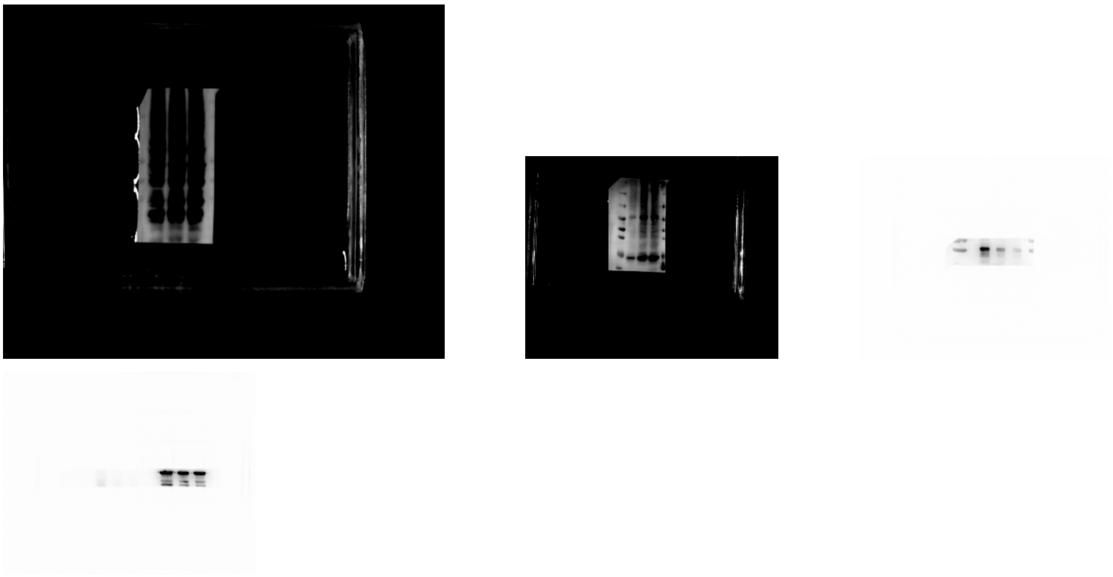

Figure 3.D

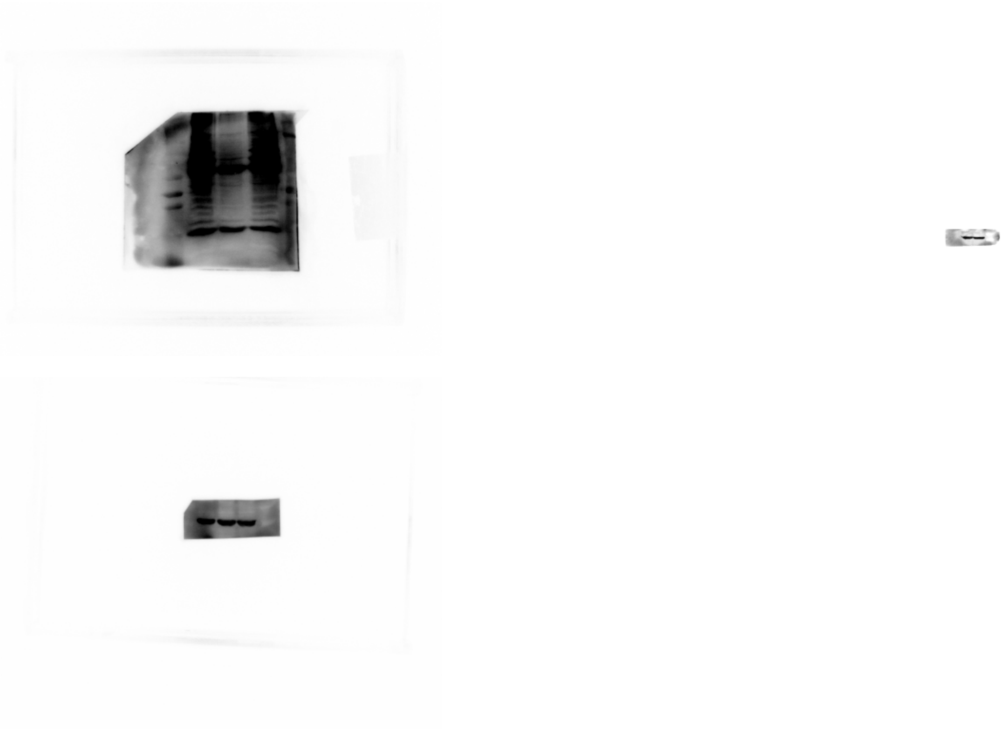

Figure 4.A

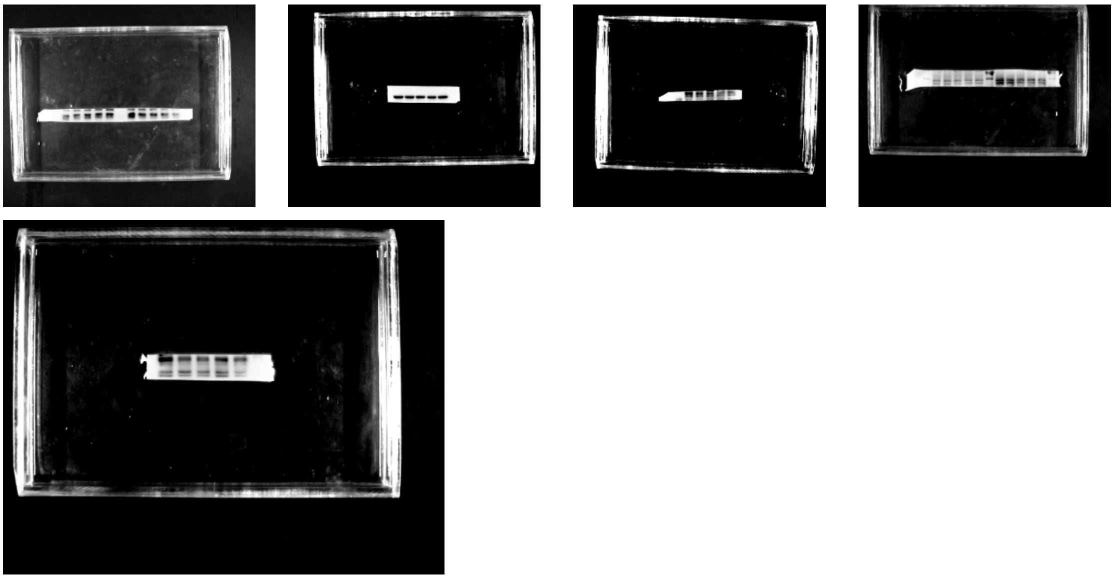

Figure 4.D

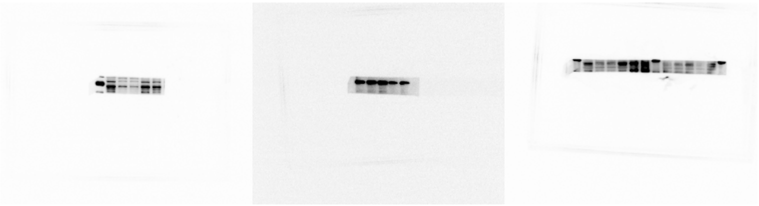

Figure 5.A,D

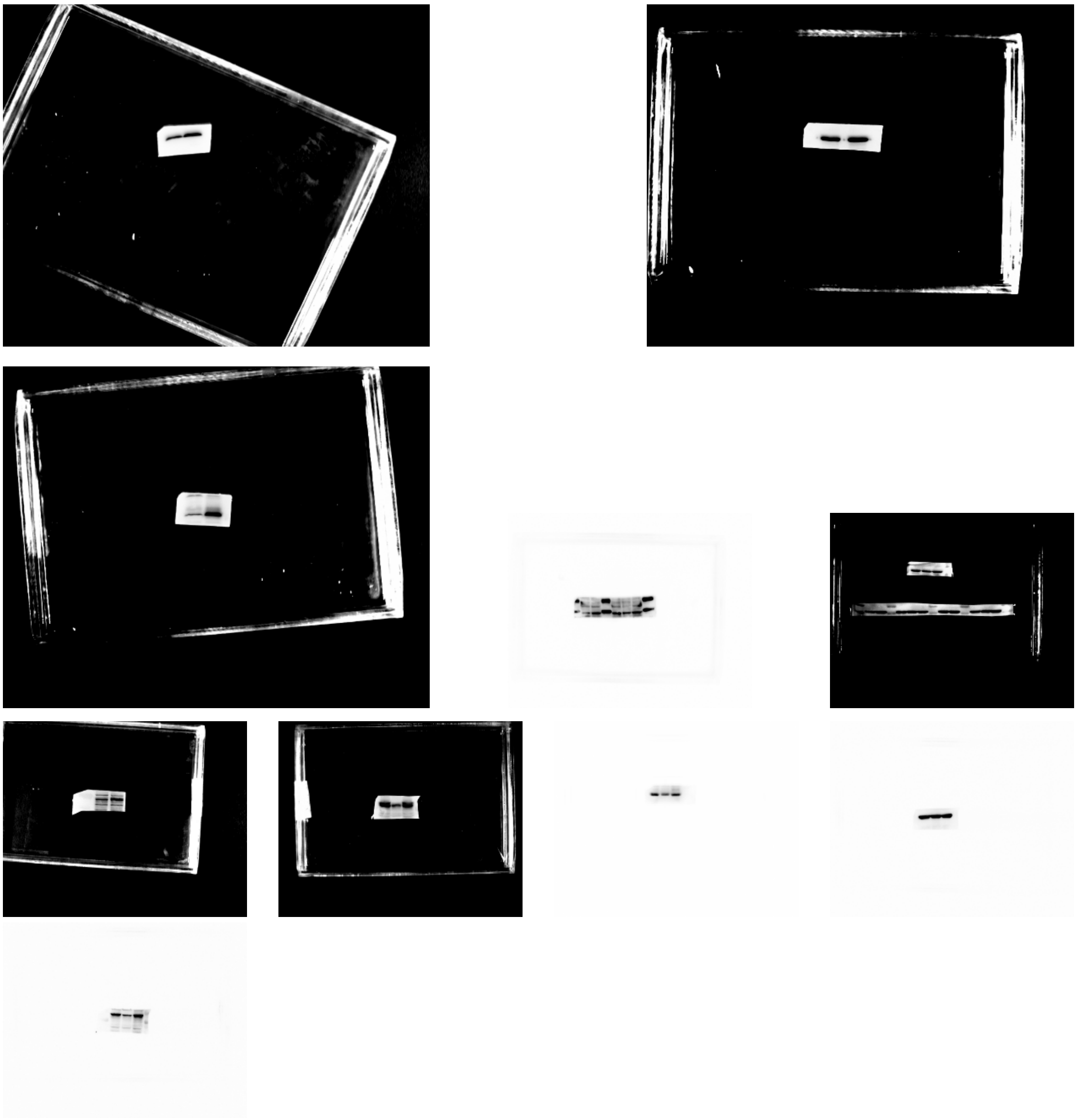

Figure 6.B

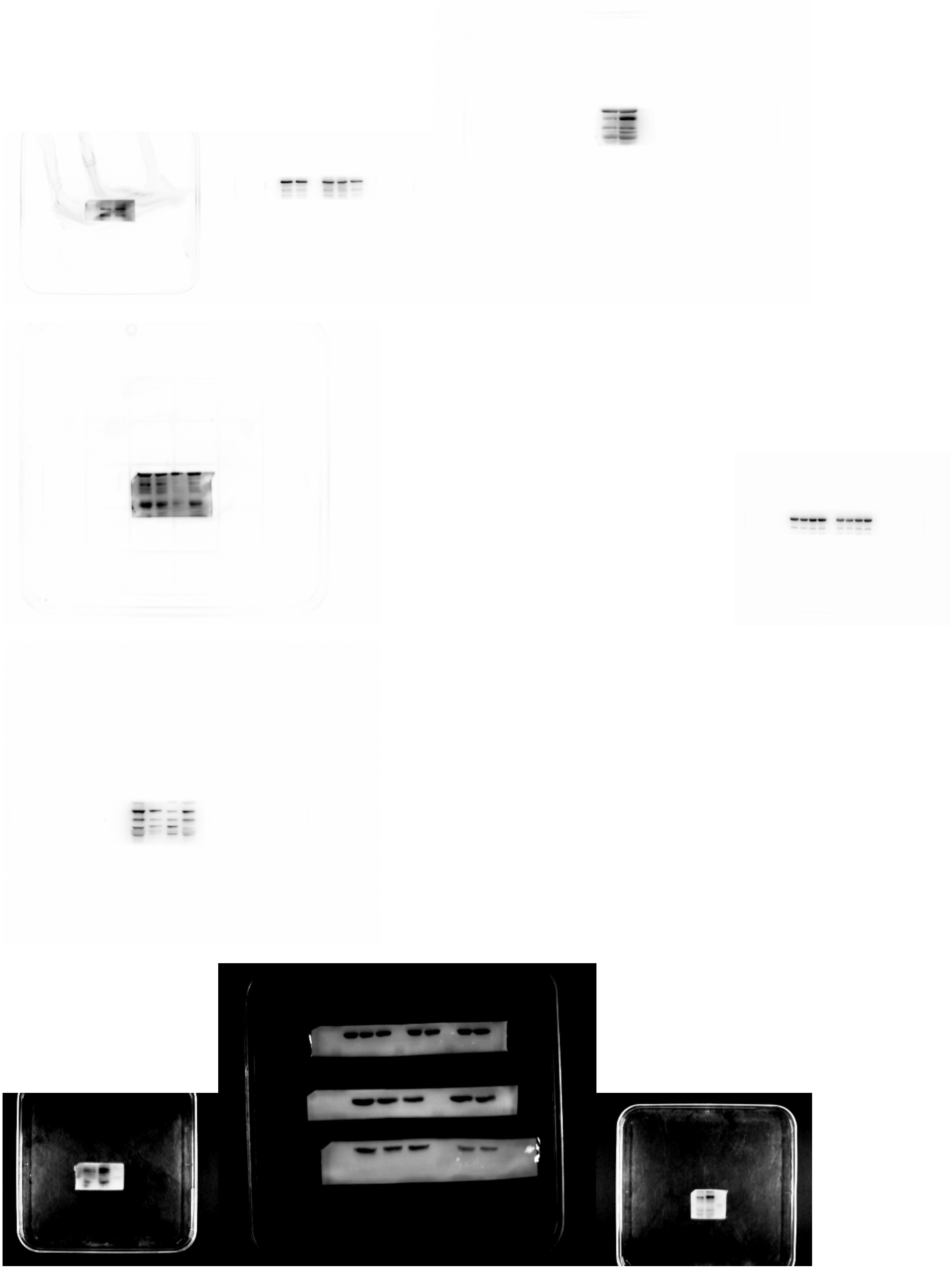

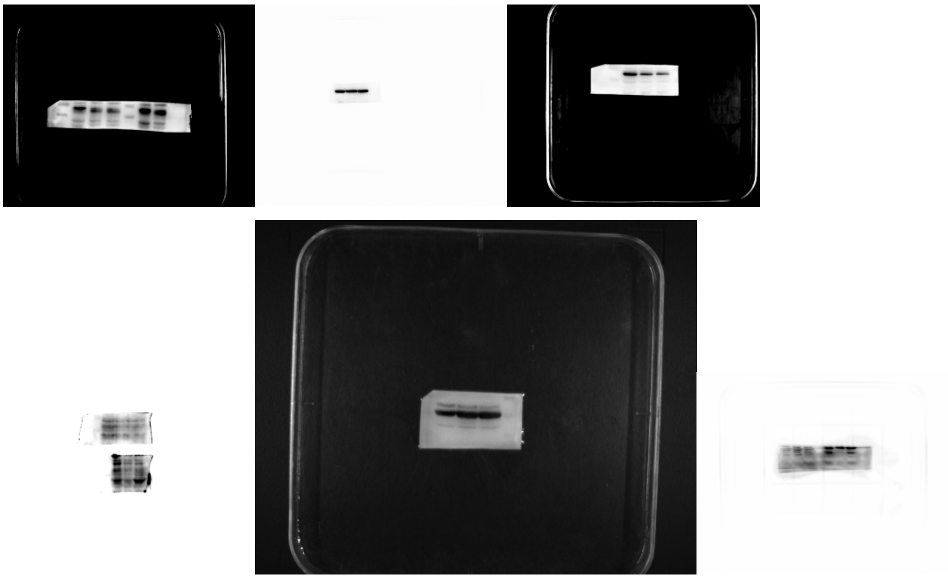

Supplementary figure.C

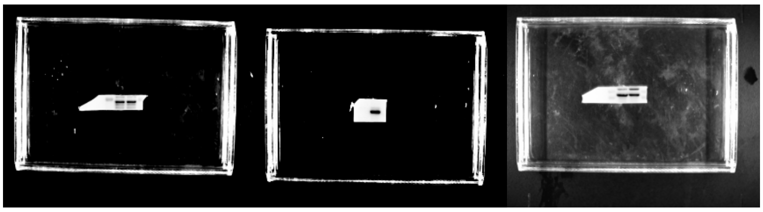

Supplementary figure.D

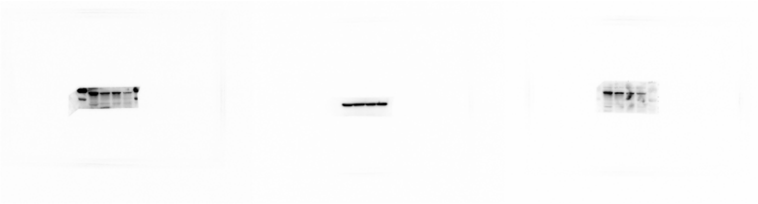

Supplementary figure.E

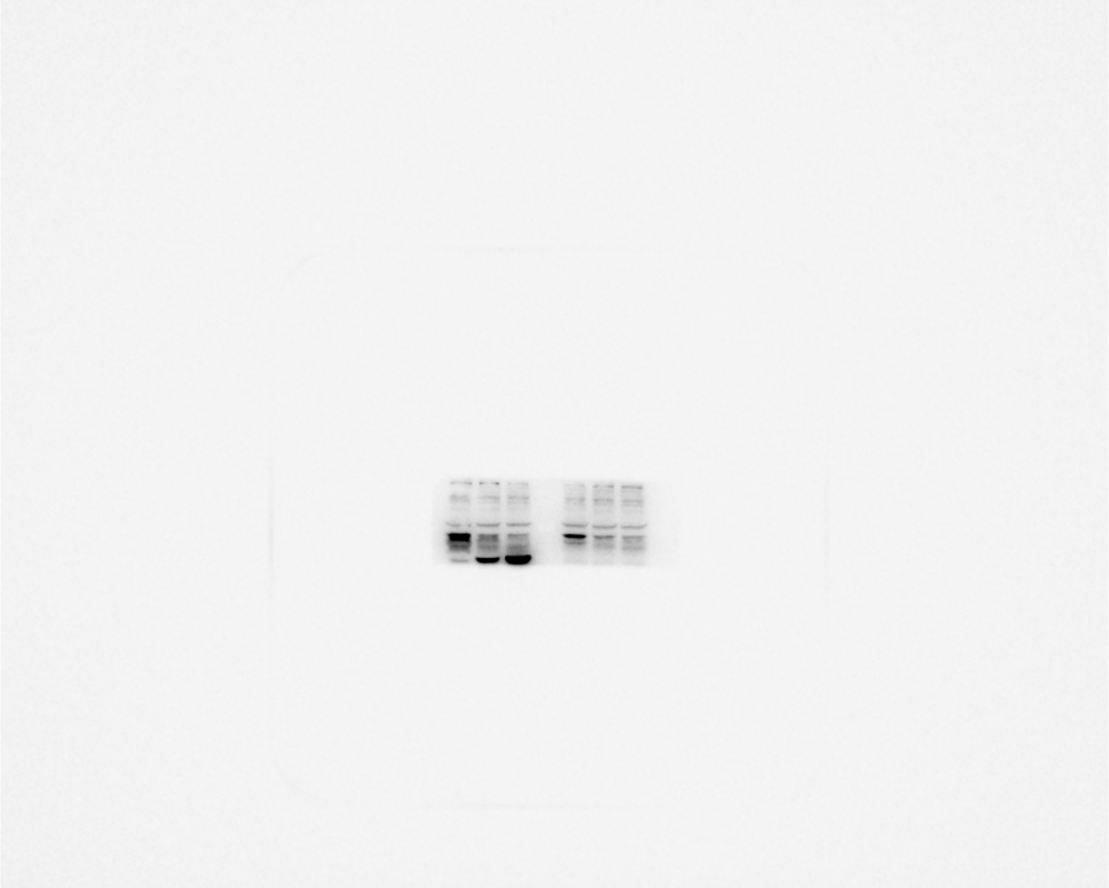

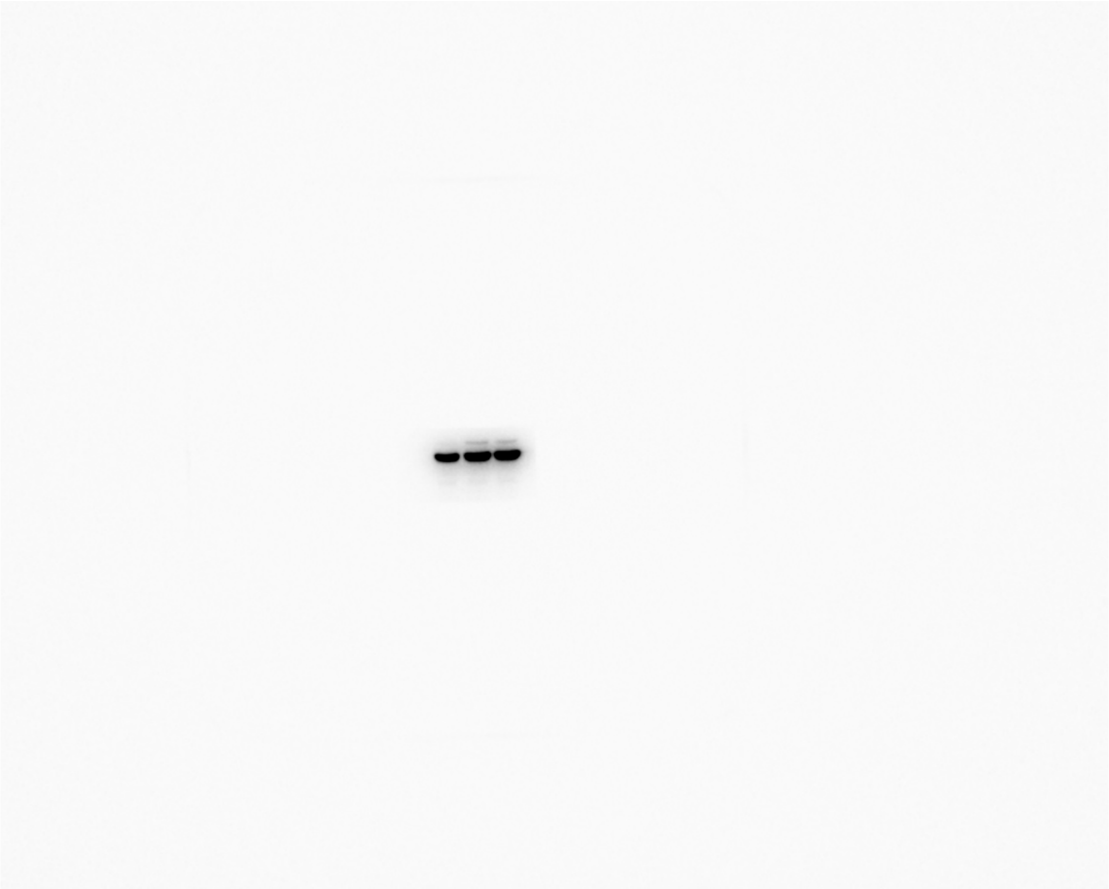

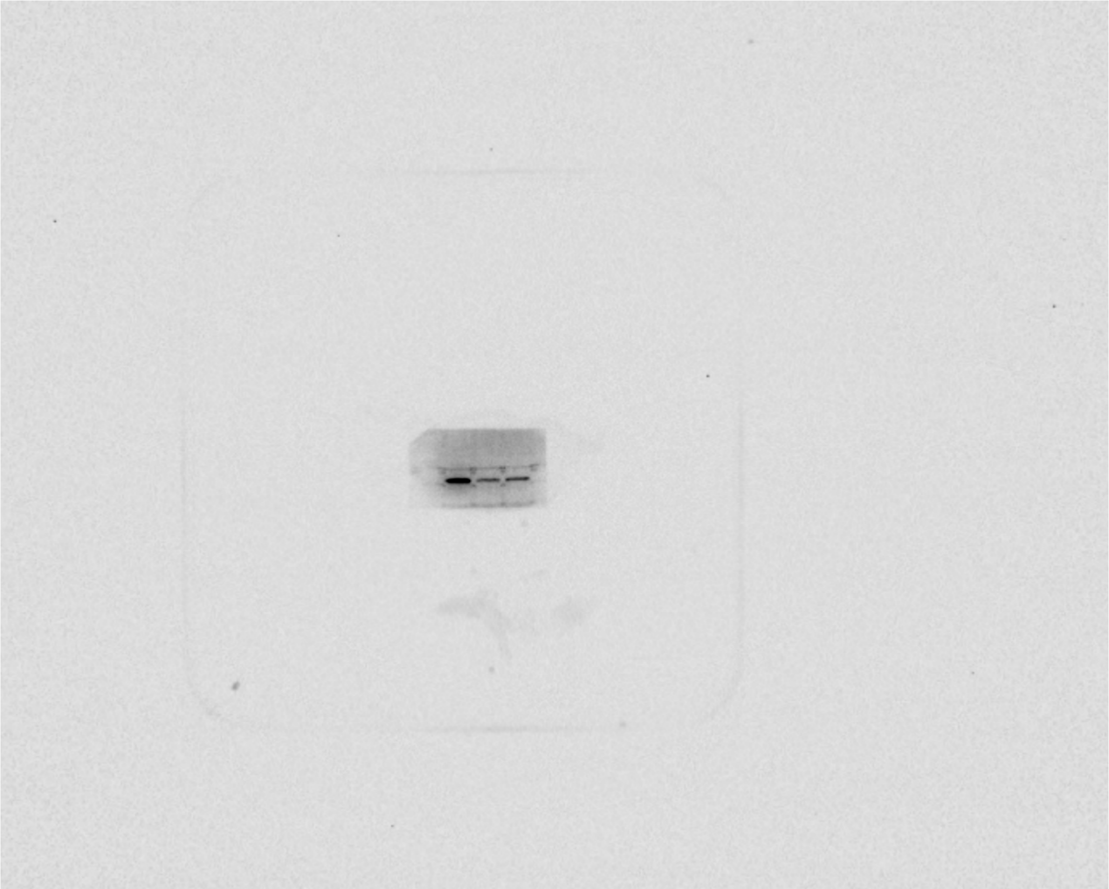

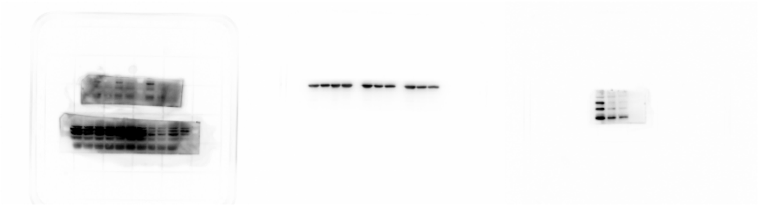

Supplement: Supplementary file 2 — western blot original data [file 41420_2022_988_MOESM2_ESM.pdf]
