# Supplementary material for: DUB3/KLF4 combats tumor growth and chemoresistance in hepatocellular carcinoma
Source: Cell Death Discov. 2022 Apr 5;8:166. doi: 10.1038/s41420-022-00988-5 (PMC8983766; doi:10.1038/s41420-022-00988-5)

上海市第一人民医院动物伦理委员会伦理审查批件

Shanghai First People's Hospital Clinical Center Laboratory Animal Welfare & Ethics Committee Approval Letter

IACUC 号：2021AWS0148

<table>
<tr><td>项目名称
(Protocol Title)</td><td colspan="3">去泛素化酶 DUB3-KLF4 通路作为抑制肝癌细胞生长新靶点的机制研究与靶向药物筛选</td></tr>
<tr><td>项目类型
(Protocol Type)</td><td colspan="3">创新药物研发</td></tr>
<tr><td>审查方式和时间
(Review Channel and Date)</td><td colspan="3">☐会议审查（full board review） ____年____月____日
☑快速审查（expedited review） 2021 年 03 月 22 日
☐紧急会议审查（emergency meeting review） ____年____月____日</td></tr>
<tr><td>审查类型
(Review Category)</td><td colspan="3">☐初始审查（Initial Review）
☐修正案审查（Review of Protocol Amendment）
☑复审（Review of Revised Protocol）
☐其他（others）</td></tr>
<tr><td>项目负责人（PI）</td><td>李琦</td><td>科室（Department）</td><td>肿瘤科</td></tr>
<tr><td>专业（Specialty）</td><td>消化道肿瘤</td><td>职称（Title）</td><td>主任医师</td></tr>
<tr><td>伦理委员会决议
(Committee Decision and Recommendation)</td><td colspan="3">审查结果（Result）：
☑同意（Approval）
☐作必要修改后同意（Approval with Recommended Revision）
☐作必要修改后重审（Resubmit after Revision）
☐不同意（Disapproval）

经审查，该项目的实验研究方案符合实验动物福利和伦理要求，同意申报。

伦理委员会主任（盖章）：
Signature of EC Chairman
上海市第一人民医院实验动物伦理委员会
2021 年 03 月 29 日</td></tr>
</table>

Mycoplasma test

Mycoplasma detection report

Sample Information

number of samples:

Customer Sample Number	Company Number
293T	20220220-01
HEPG2	20220220-02
OVCAR8	20220220-03
HEP3B	20220220-04
LO2	20220220-05
Huh7	20220220-06

Sample quantity: 6

Characteristics of sample: cell culture medium, cell precipitation

Detection items: Mycoplasma detection

Inspection department :

Detection methods: PCR was used to amplify specific sequences of Mycoplasma, and agarose electrophoresis was used to analyze the results.

Experimental Process

(1) Sample preparation

Sample type: culture supernatant

The 1ml culture supernatant sample was taken into a clean and sterilized 1.5ml centrifuge tube, and the precipitation was collected by centrifugation at 13000rpm.

An appropriate amount of lysate was added and treated at 95℃ for 5min, and the supernatant was retained by centrifugation for detection.

Sample type: cell precipitation

An appropriate amount of lysate was used to treat the cell precipitates, and the supernatant was retained by centrifugation for detection.

(2) Experimental system

PCR system (unit: UL)	negative	positive	20220220-01 detector tube	20220220-02 detector tube	20220220-03 detector tube	20220220-04 detector tube	20220220-05 detector tube	20220220-06 detector tube
PCR-Mix	18	18	18	18	18	18	18	18
Negative control	2	-	-	-	-	-	-	-
Positive Control	-	2	-	-	-	-	-	-
Sample DNA	-	-	2	2	2	2	2	2
Total volume	20	20	20	20	20	20	20	20

According to the above system configuration, PCR reaction detection was carried out.

(3) Circular procedures

50℃2min	A loop
95℃10min	A loop
95℃30sec	35 cycles
60℃45sec	
72℃45sec	
72℃ 5min	

PCR reaction procedures were set up according to the above cycles. The products were electrophoretized in 3% agarose gel. PCR products and maker were sampled for 5ul, and were observed and photographed by gel imager.

Test Results

(1) Experimental results

Electrophoresis results are shown as follows:

pos. neg.

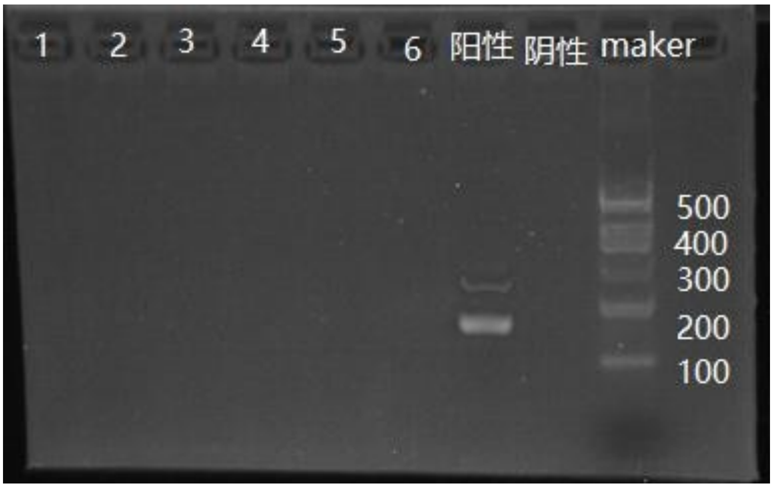

In the figure, lane 1 is the detection product of 20220306-01, and lane 2 is the detection product of 20220306-02. 3 is the detection product 20220306-03. 4 is the detection product 20220306-04. 5 is the detection product of 20220306-05. 6 is the detection product of 20220306-06. The negative PCR products had no bands, and the positive PCR products had 280bp and 150bp bands, indicating that the experiment was successful, and the detected products only had 150bp bands

Or no bands indicated no mycoplasma contamination, 280bp and 150bp bands indicated mycoplasma contamination, and only 280bp bands indicated serious mycoplasma contamination.

(2) Conclusion

Sample name	mycoplasma contamination
293T	Neg.
HEPG2	Neg.
OVCAR8	Neg.
HEP3B	Neg.
LO2	Neg.
Huh7	Neg.

Identification and detection of cell genetic quality

Cell Line Authentication Service

STR Genotyping test report

Sample Information

Number of samples：

Customer Sample Number	Company Number
HEPG2	20200621-01

Sample Quantity: 1

Sample properties: Cell line

Test Item: STR

Department of detection: Fu heng

Methods: DNA was extracted with Axygen genome extraction kit and amplified with 21-str amplification scheme. STR loci and sex gene Amelogenin were detected on ABI 3730XL genetic analyzer.

Test Results

(1) Check the basic situation

Sample number	Company No.	Multiple alleles	Matched cell line	Cell bank	Exposure value	Match
HEPG2	20200621-01	N	HEP-3B	DSMZ	0.97	Yes

Genotypic test results of samples

- A multi-allele refers to a phenomenon involving genes at or above the third isotonic location。
- According to the method, the cell typing results were good.

(2) Description of each sample

20200621-01: Cell DNA typing of this strain found a basically matched cell line in cell line retrieval. DSMZ database showed that the cell name was HEP-G2 and the cell number was corresponding to ACC-180. No multiple alleles were found in this cell line.

Note: Cell lines to be tested were compared with STR data in ATCC, DSMZ, JCRB and RIKEN databases. Cell lines not included in the above database will not be matched.

(3) Sample typing results

Genotyping results of STR locus and Amelogenin locus in cells						
Loci	STR information of sent cells			STR information of cell bank		
	Cell name:HEPG2			Cell bank Cell Name:HEP-G2		
	Allele1	Allele2	Allele3	Allele1	Allele2	Allele3
D5S818	11	12		11	12	
D13S317	9	13		9	13	
D7S820	10	10		10	10	
D16S539	12	12		12	13	
VWA	17	17		17	17	
TH01	9	9		9	9	
AMEL	X	Y		X	Y	
TPOX	8	9		8	9	
CSF1PO	10	11		10	11	
D12S391	21	25				
FGA	22	25				
D2S1338	19	20				
D21S11	29	31				
D18S51	13	14				
D8S1179	15	16				
D3S1358	15	16				
D6S1043	13	13				
PENTAE	15	20				
D19S433	15.2	15.2				
PENTAD	9	13				
D1S1656	11	12				

Other Description

(1) Classification scheme and locus distribution

	Plan 1	Plan 2	Plan 3	Plan 4
1	D3S1358	D8S1179	D19S433	AMEL
2	VWA	D21S11	TH01	D1S1656
3	D7S820	D16S539	D13S317	D5S818
4	CSF1PO	D2S1338	TPOX	D12S391
5	PENTAE	PENTAD	D18S51	FGA
6			D6S1043	

Experimental scheme and site

(2) STRdatabasecomparison

We used DSMZ Tools for cell line comparison, which included 2455 cell line STR data from ATCC, DSMZ, JCRB and RIKEN databases. If the cells to be detected are not included in the above cell bank or this is a new cell line established by the user, it will not be able to be compared with other databases according to the cell typing results.

Applied Biosystems
GeneMapper ID v3.2

20210924

Sample File	Sample Name	Panel	SQO	OS	SQ
73_A10_CellLineAuthentication-1-0926.fsa	Hep3B	21Plex_STR_Panel_v0.4			

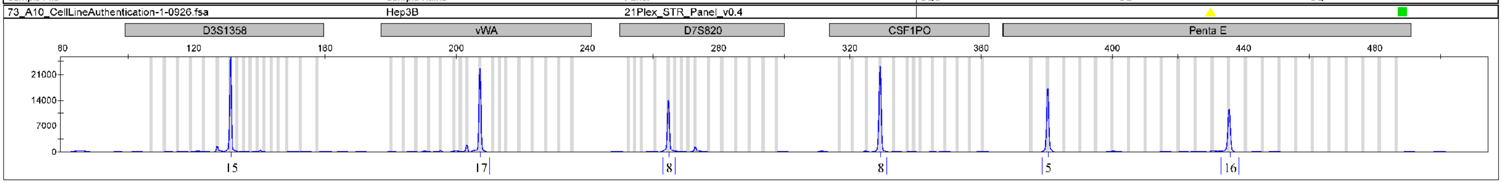

Sample File	Sample Name	Panel
73_A10_CellLineAuthentication-1-0926.fsa	Hep3B	21Plex_STR_Panel_v0.4

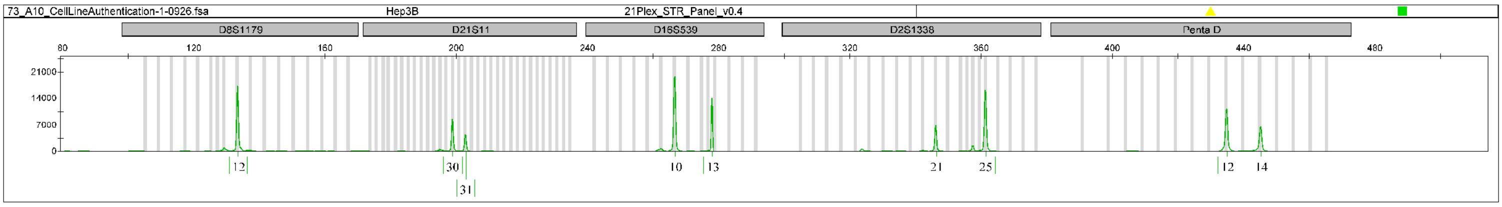

Sample File	Sample Name	Panel
73_A10_CellLineAuthentication-1-0926.fsa	Hep3B	21Plex_STR_Panel_v0.4

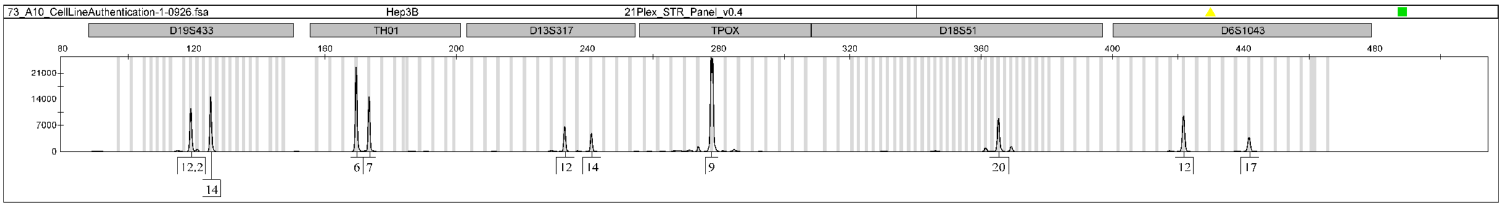

Sample File	Sample Name	Panel
73_A10_CellLineAuthentication-1-0926.fsa	Hep3B	21Plex_STR_Panel_v0.4

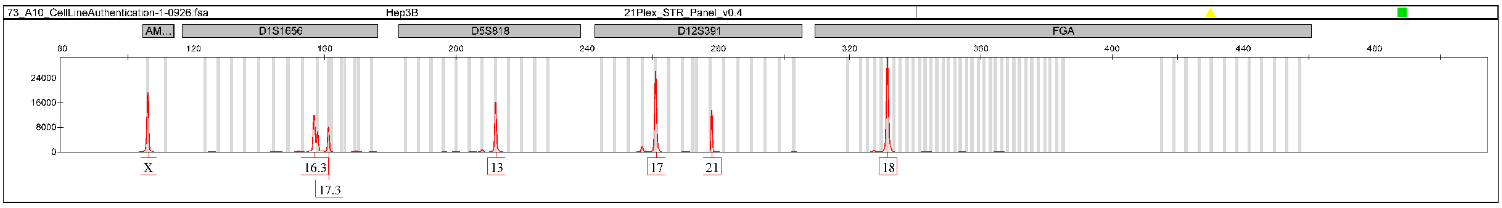

Identification and detection of cell genetic quality

Cell Line Authentication Service

STR Genotyping test report

Sample Information

Number of samples：

Customer Sample Number	Company Number
HEP3B	20210924-01

Sample Quantity: 1

Sample properties: Cell line

Test Item: STR

Department of detection: Fu heng

Methods: DNA was extracted with Axygen genome extraction kit and amplified with 21-str amplification scheme. STR loci and sex gene Amelogenin were detected on ABI 3730XL genetic analyzer.

Test Results

(1) Check the basic situation

Sample number	Company No.	Multiple alleles	Matched cell line	Cell bank	Exposure value	Match
HEP3B	20210924-01	N	HEP-3B	DSMZ	0.94	Yes

Genotypic test results of samples

- A multi-allele refers to a phenomenon involving genes at or above the third isotonic location。
- According to the method, the cell typing results were good.

(2) Description of each sample

20210924-01: Cell DNA typing of this strain found a basically matched cell line in cell line retrieval. DSMZ database showed that the cell name was HEP-3B and the cell number was corresponding to ACC-93. No multiple alleles were found in this cell line.

Note: Cell lines to be tested were compared with STR data in ATCC, DSMZ, JCRB and RIKEN databases. Cell lines not included in the above database will not be matched.

(3) Sample typing results

Genotyping results of STR locus and Amelogenin locus in cells						
Loci	STR information of sent cells			STR information of cell bank		
	Cell name:HEP3B			Cell bank Cell Name:HEP-3B		
	Allele1	Allele2	Allele3	Allele1	Allele2	Allele3
D5S818	13	13		13	13	
D13S317	12	14		12	14	
D7S820	8	8		8	10	
D16S539	10	13		10	10	
VWA	17	17		17	17	
TH01	6	7		6	7	
AMEL	X	X		X	X	
TPOX	9	9		9	9	
CSF1PO	8	8		8	8	
D12S391	17	21				
FGA	18	18				
D2S1338	21	25				
D21S11	30	31				
D18S51	20	20				
D8S1179	12	12				
D3S1358	15	15				
D6S1043	12	17				
PENTAE	5	16				
D19S433	12.2	14				
PENTAD	12	14				
D1S1656	16.3	17.3				

Other Description

(1) Classification scheme and locus distribution

	Plan 1	Plan 2	Plan 3	Plan 4
1	D3S1358	D8S1179	D19S433	AMEL
2	VWA	D21S11	TH01	D1S1656
3	D7S820	D16S539	D13S317	D5S818
4	CSF1PO	D2S1338	TPOX	D12S391
5	PENTAE	PENTAD	D18S51	FGA
6			D6S1043	

Experimental scheme and site

(2) STRdatabasecomparison

We used DSMZ Tools for cell line comparison, which included 2455 cell line STR data from ATCC, DSMZ, JCRB and RIKEN databases. If the cells to be detected are not included in the above cell bank or this is a new cell line established by the user, it will not be able to be compared with other databases according to the cell typing results.

Applied Biosystems
GeneMapper ID v3.2

20210924

Sample File	Sample Name	Panel	SQO	OS	SQ
73_A10_CellLineAuthentication-1-0926.fsa	Hep3B	21Plex_STR_Panel_v0.4			

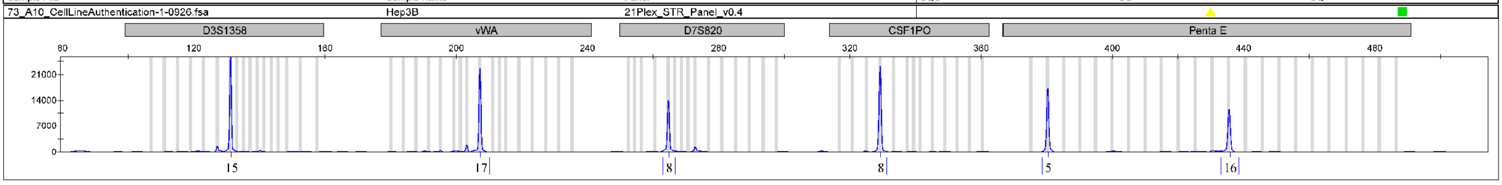

73_A10_CellLineAuthentication-1-0926.fsa Hep3B 21Plex_STR_Panel_v0.4

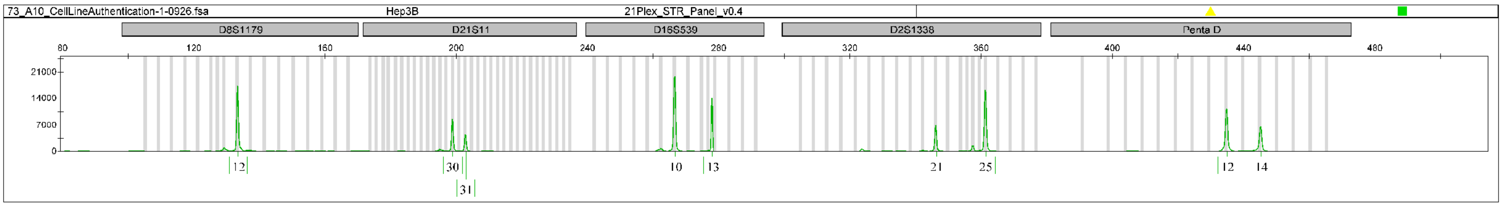

73_A10_CellLineAuthentication-1-0926.fsa Hep3B 21Plex_STR_Panel_v0.4

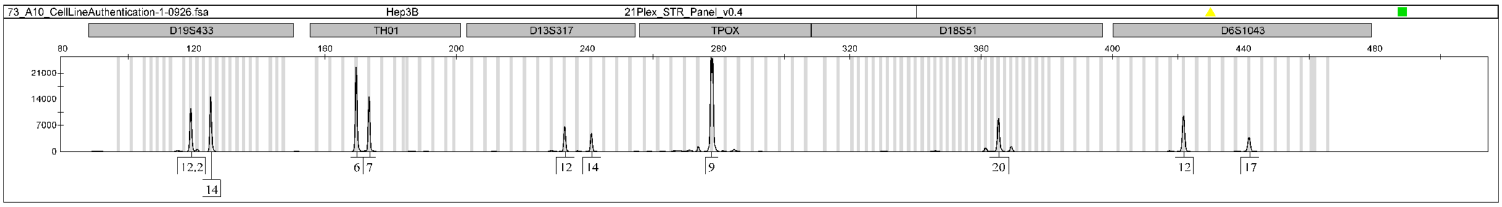

73_A10_CellLineAuthentication-1-0926.fsa Hep3B 21Plex_STR_Panel_v0.4

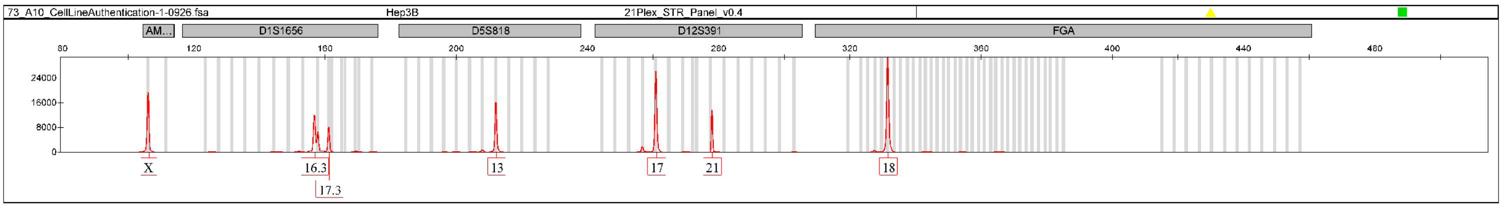

Supplement: Supplementary file 3 — Manuscript Related File [file 41420_2022_988_MOESM3_ESM.pdf]
